# Lemon Juice-Assisted Green Extraction of Strawberry Enhances Neuroprotective Phytochemicals: Insights into Alzheimer’s-Related Pathways

**DOI:** 10.3390/ph18121892

**Published:** 2025-12-15

**Authors:** Youssef Mohamed Sharaf, Jilan A. Nazeam, Karema Abu-Elfotuh, Ayah M. H. Gowifel, Ahmed M. Atwa, Ehsan Khedre Mohamed, Ahmed M. E. Hamdan, Reema Almotairi, Amira M. Hamdan, Samir M. Osman, Hala M. El Hefnawy

**Affiliations:** 1 Pharmacy Department, National Cancer Institute, Cairo University, Cairo 11562, Egypt; 2 Pharmacognosy Department, Faculty of Pharmacy, October 6 University, Giza 12566, Egypt; 3 Clinical Pharmacy Department, Faculty of Pharmacy (Girls), Al-Azhar University, Cairo 11884, Egypt; 4 College of Pharmacy, Al-Ayen Iraqi University (AUIQ), An Nasiriyah 64001, Iraq; 5 Pharmacology and Toxicology Department, Faculty of Pharmacy, Modern University for Technology and Information (MTI), Cairo 11571, Egypt; 6 Pharmacology and Toxicology Department, Faculty of Pharmacy, Egyptian Russian University, Badr, Cairo 11829, Egypt; 7 Department of Biochemistry, Egyptian Drug Authority (EDA), Formerly National Organization of Drug Control and Research (NODCAR), Giza 12112, Egypt; 8 Department of Pharmacology and Toxicology, Faculty of Pharmacy, University of Tabuk, Tabuk 71491, Saudi Arabia; a_hamdan@ut.edu.sa; 9 Prince Fahad bin Sultan Chair for Biomedical Research (PFSCBR), Tabuk 74191, Saudi Arabia; 10 Department of Medical Laboratory Technology, Faculty of Applied Medical Sciences, Prince Fahad Bin Sultan Chair for Biomedical Research, University of Tabuk, Tabuk 71491, Saudi Arabia; 11 Faculty of Science, Alexandria University, Alexandria 21511, Egypt; 12 Pharmacognosy Department, Faculty of Pharmacy, Cairo University, Cairo 11562, Egypt

**Keywords:** Acid-assisted extraction, Alzheimer’s disease, green extraction, lemon juice, natural products, oxidative stress, strawberry extract, Wnt3/β-catenin signaling

## Abstract

**Background/Objective**: Alzheimer’s disease (AD) is a neurodegenerative condition characterized by oxidative stress, neuroinflammation, amyloidogenesis, and tau-related pathology. This study investigated the macronutrient and phytochemical composition of strawberry (S), lemon (L), and lemon juice-assisted strawberry (S/L) extracts and evaluated their neuroprotective efficacy relative to selenium (Se) in an aluminum chloride (AlCl_3_)-induced rat model of AD. **Methods**: Macronutrients and phenolics were quantified in S, L, and S/L, and the extracts were profiled using high-performance liquid chromatography and electrospray ionization tandem mass-spectrometry. Male Sprague–Dawley rats received AlCl_3_ with or without S, L, S/L, or Se, and their cognitive performance was assessed using the Morris water maze, Y-maze, and conditioned avoidance tests. Markers of oxidative status, inflammation, cholinergic function, apoptotic signaling, and Wnt3/β-catenin pathway activity were quantified in the brain tissue, and cortico-hippocampal morphology was examined. **Results**: The S/L extract showed the highest carbohydrate, protein, and lipid content. The total phenolic content was highest in S/L (60.46 mg gallic acid equivalents/g), followed by L (55.08) and S (44.75), with S/L also being the richest in gallic, ellagic, and chlorogenic acids. S/L attenuated AlCl_3_-induced cognitive deficits, restored antioxidant status, suppressed neuroinflammation, improved cholinergic indices, modulated apoptotic signaling, and downregulated amyloidogenic and *NLRP3* inflammasome markers, consistent with histological evidence of neuronal preservation. **Conclusions**: Lemon juice-assisted extraction enhanced the macronutrient and phenolic richness and multitarget neuroprotection of strawberries. S/L co-extracts represent promising functional food–derived adjuvants for AD management and support integrative compositional–mechanistic profiling to optimize natural product–based interventions.

## 1. Introduction

Alzheimer’s disease (AD), the most prevalent form of dementia, is a progressive and irreversible neurodegenerative disorder that poses substantial global health and economic challenges. According to estimates from the World Health Organization (WHO), over 55 million individuals currently live with dementia, a figure projected to rise to 78 million by 2030 and 139 million by 2050, with the associated economic burden expected to surpass $2.8 trillion [1,2]. The pathophysiology of AD is multifaceted and is characterized by progressive cognitive decline, memory impairment, neuroinflammation, oxidative stress, and pathological accumulation of amyloid-β (Aβ) plaques and neurofibrillary tangles (NFTs). These hallmarks contribute to synaptic dysfunction, mitochondrial impairment, and eventual neuronal death in the brain [3].

The deposition of Aβ plaques and NFTs in the brain is associated with disrupted synaptic communication, mitochondrial dysfunction, and activation of neuroinflammatory cascades, ultimately resulting in neurodegeneration [4,5]. As the disease advances, patients often experience severe complications, such as thrombosis, dysphagia, malnutrition, impaired mobility, and pneumonia, all of which contribute to increased morbidity and mortality [6,7]. Although significant research has been conducted, the precise etiology of AD remains unclear [8]. Clinically, AD is characterized by progressive cognitive deterioration, including memory loss, neuropsychiatric symptoms, and impairments in visual and language processing [9,10].

Current pharmacological treatments, such as donepezil [11], rivastigmine [12], galantamine [13], memantine [14], and namzaric [15], primarily provide symptomatic relief, with limited impact on disease progression [16]. Moreover, these therapies are frequently associated with adverse effects and declining efficacy over time [17]. Consequently, there is a critical need for alternative or adjunctive therapeutic strategies that target the underlying neuropathological mechanisms of AD while offering improved safety and sustainability [2,18].

Dietary and nutraceutical interventions have garnered increasing attention for their potential to mitigate cognitive decline and neurodegenerative processes [19,20]. Nutritional status is now recognized as a key modulator of aging and neurological health, and various bioactive food-derived compounds have demonstrated promising neuroprotective properties [21]. Phenolic and flavonoid compounds, in particular, have attracted significant interest because of their antioxidant, anti-inflammatory, and anti-amyloidogenic activities [22,23]. Emerging evidence underscores the capacity of antioxidants to counteract neuroinflammation and oxidative stress, providing mechanistic justification for their potential role in Alzheimer’s disease therapy [24].

Citrus limon (lemon) is a citrus fruit rich in secondary metabolites, such as limonene and its derivatives, which exhibit neuroprotective, antioxidative, and anti-inflammatory properties [25]. These compounds are implicated in the attenuation of mitochondrial dysfunction and neuronal stress pathways, which are central to AD pathogenesis [26]. Additionally, lemon juice has been shown to improve memory and cognitive function in preclinical models of scopolamine-induced amnesia, possibly through the upregulation of hippocampal ERK and *β-actin* signaling [27]. Beyond its pharmacological effects, lemon juice serves as a natural, food-grade acidifying agent that can enhance the extraction efficiency of polyphenolic compounds from plant matrices, providing a sustainable alternative to synthetic mineral acids [28,29,30].

Strawberry (*Fragaria ananassa*), a widely cultivated member of the Rosaceae family, is a rich source of diverse phytochemicals, including proanthocyanidins, anthocyanins (e.g., pelargonidin, cyanidin), ellagic acid, flavonols, catechins, phenolic acids (chlorogenic, caffeic), carotenoids, and vitamins C and E [31]. These constituents confer a broad spectrum of biological activities, such as antioxidant, cardioprotective, antihypertensive, and anti-inflammatory activities, and are used in the management of esophageal cancer [32]. Several studies have indicated that strawberries exert neuroprotective effects by mitigating oxidative stress, suppressing neuroinflammatory pathways, and reducing Aβ accumulation [32,33,34]. Given the limited availability of disease-modifying treatments for AD, strawberries offer a safe, accessible, and cost-effective dietary intervention. Epidemiological evidence indicates that individuals who consume strawberries more than once per week exhibit a 32% lower risk of developing AD than non-consumers [35]. Furthermore, randomized controlled trials have demonstrated that dietary supplementation with freeze-dried strawberries (24 g/day, equivalent to two cups of fresh fruit) significantly enhances cognitive performance in older adults [36].

Despite the promising individual neuroprotective profiles of strawberries and lemons, their combined efficacy and potential synergistic effects have not been adequately explored. Additionally, the use of lemon juice-assisted extraction (LJAE) as a green extraction method to optimize the yield and bioactivity of strawberry phenolics has not been systematically investigated. Furthermore, a persistent limitation of the current literature is the lack of chemically standardized extracts and incomplete elucidation of the molecular mechanisms underlying their neuroprotective actions.

Accordingly, the present study aimed to evaluate the neuroprotective potential of strawberry extract (S), lemon juice (L), and their combination (S/L) in an aluminum chloride (AlCl_3_)-induced rat model of AD. Specifically, this study aimed to (i) assess the synergistic effects of co-supplementation on behavioral, biochemical, and histopathological endpoints relevant to AD; (ii) characterize the phytochemical composition of the extracts using high-performance liquid chromatography (HPLC) and LC-ESI-MS/MS; (iii) investigate the efficacy of lemon juice as a natural acidifying agent in enhancing phenolic yield and bioactivity; and (iv) elucidate the underlying molecular mechanisms, with a focus on signaling pathways related to oxidative stress, inflammation, and amyloidogenesis. This integrative approach is intended to establish a foundation for functional food-based interventions in the management of Alzheimer’s disease.

## 2. Results

### 2.1. Phytochemical Profiling of Extracts

#### 2.1.1. Macronutrient Profiling

The three extracts exhibited distinct macronutrient profiles. The Strawberry/Lemon extract contained the highest concentration of total carbohydrates at 9.98 mg/g, followed by the strawberry extract with 8.23 mg/g, whereas the lemon extract had the lowest carbohydrate content at 1.04 mg/g. Total protein was nearly undetectable in the strawberry extract (0.01 mg/g), but was present at substantially higher levels in both the lemon (0.69 mg/g) and strawberry/lemon (0.71 mg/g) extracts. Similarly, the total lipid content was lowest in the strawberry extract (0.25 mg/g) and highest in the combined strawberry-lemon extract (4.58 mg/g), with the lemon extract containing an intermediate amount of lipids (3.27 mg/g).

**Figure 1:**
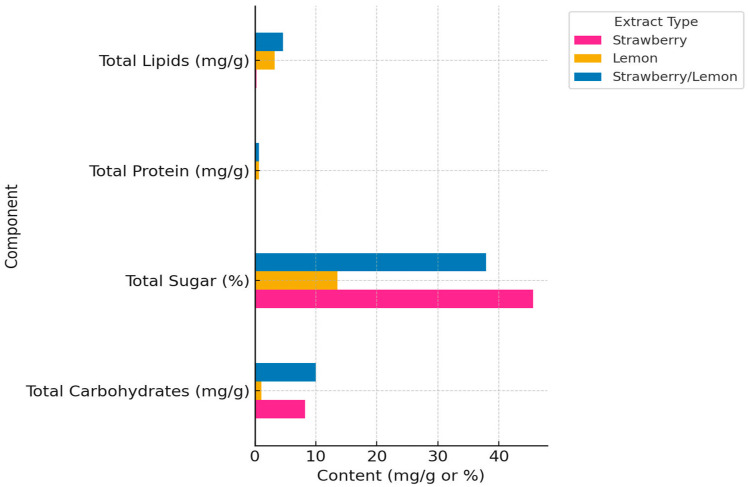
Comparative macronutrient composition of Strawberry, Lemon, and Strawberry/Lemon extracts. The horizontal bar chart illustrates the levels of total carbohydrates (mg/g), total sugars (%), total proteins (mg/g), and total lipids (mg/g) in the three extract types. The data highlight significant differences in nutrient distribution, with strawberry–lemon extract exhibiting the highest lipid and carbohydrate contents, while strawberry extract shows the highest sugar content. The measurements were performed under rigorously standardized analytical conditions.

Strawberry extract contained the highest sugar content, with total sugars reaching 456.3 mg/g dry weight (DW) and reducing sugars reaching 385.7 mg/g DW. In contrast, lemon extract showed markedly lower sugar levels, with total sugars of 135.4 mg/g DW and reducing sugars of 98.3 mg/g of DW. The combined strawberry/lemon extract exhibited intermediate values (378.6 mg/g DW total sugars and 259.5 mg/g DW reducing sugars), lower than strawberry alone but substantially higher than lemon, indicating that the high sugar content of strawberry was partially diluted in the S/L extract while remaining detectable (Figure 1).

#### 2.1.2. Quantitative Determination of Phenolics Content

**Figure 2:**
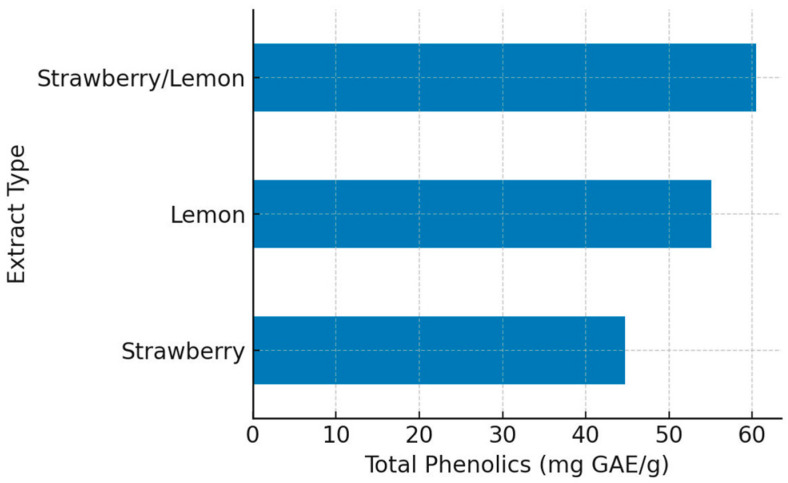
Comparative bar chart representing the levels of total phenolics (mg gallic acid/gm) in different extracts: strawberry (S), lemon (L), and strawberry/lemon (S/L).

The total phenolic content in the extracts was measured in milligrams of gallic acid equivalents per gram (mg GAE/g). The S/L extract exhibited the highest phenolic content at 60.46 mg GAE/g, surpassing the lemon (L) (55.08 mg GAE/g) and strawberry (S) (44.75 mg GAE/g) extracts. Notably, the use of lemon juice as a coextractor of strawberry extract resulted in a greater abundance of phenolic compounds (Figure 2). HPLC analysis revealed distinct differences in phenolic content among the S, L, and S/L extracts. The S/L extract had the highest concentrations of gallic acid (5301.79 µg/g), ellagic acid (214.58 µg/g), and chlorogenic acid (4558.92 µg/g), indicating that lemon juice-assisted extraction significantly enhanced the bioactive yield compared to the individual extracts (Table S1, Supplementary File). This study elucidated the substantial variations in the phytochemical compositions of strawberry extract, lemon juice, and lemon juice-assisted strawberry extract. These insights are valuable for optimizing extraction methods to increase the yield of bioactive metabolites in functional food applications.

### 2.2. LC-ESI-MS/MS Profiling

Phytochemical characterization of strawberry (S), lemon (L), and lemon juice-assisted strawberry extracts (S/L) was performed using LC–ESI–MS/MS in the negative ionization mode. Compound identification was achieved based on retention time (RT), accurate mass (*m*/*z*), and MS/MS fragmentation patterns, referencing data from standard libraries (e.g., MassBank and METLIN) and literature.

#### 2.2.1. LC/MS/MS of Strawberry (S) Extract

Strawberry extracts contain a diverse range of polyphenols, anthocyanins, and sesquiterpenoids (Table 1, Supplementary File Table S2). Gallic acid (RT: 1.90 min, *m*/*z* 169.09 [M–H]^−^) exhibited characteristic fragmentation, yielding *m*/*z* 124.99 and 141.99, corresponding to the loss of CO_2_ (−44 Da) and hydroxyl rearrangements, respectively, which is consistent with prior studies on hydroxybenzoic acids [37]. *p*-Coumaric acid (RT: 4.72 min, *m*/*z* 163.00) displayed a fragment at *m*/*z* 119.04 due to the neutral loss of the carboxyl group. Chlorogenic acid (RT: 6.11 min, *m*/*z* 354.99) was identified based on its fragmentation at *m*/*z* 193.06 (quinic acid moiety).

Anthocyanins were also prominent, including cyanidin-3-O-hexoside (RT: 5.26 min, *m*/*z* 448.94), which fragmented to *m*/*z* 286.97 after the neutral loss of a hexose moiety (−162 Da). Pelargonidin derivatives such as pelargonidin-3-glucoside (RT: 7.04 min, *m*/*z* 415.01) and pelargonidin-3-malonylglucoside (RT: 9.19 min, *m*/*z* 518.04) demonstrated glycosidic cleavage to their aglycone (*m*/*z* 270.07). Anthocyanins contribute to the characteristic color of fruits, have strong antioxidant capabilities, and are associated with the modulation of synaptic plasticity and reduction of neuroinflammation [38].

Phenolic acids, such as gallic, *p*-coumaric, and chlorogenic acids, have also been identified as providing additional antioxidant and anti-inflammatory support [39]. Moreover, Ding et al. [40] demonstrated that gallic acid significantly alleviates cognitive impairment in an APP/PS1 mouse model of Alzheimer’s disease by promoting neurogenesis. This effect is mediated through the activation of the *GSK3β*-*Nrf2* signaling pathway, highlighting the therapeutic potential of GA in enhancing cognitive function and supporting neuronal regeneration in AD. In addition, flavonoid glycosides, including isoquercitrin and hesperidin, were present and are known to influence oxidative signaling pathways, with hesperidin notably having neurobehavioral and neuroinflammatory activities [41].

Flavonoid content was evidenced by isoquercitrin (RT: 9.52 min, *m*/*z* 464.04), which showed fragment ions at *m*/*z* 301.03 (quercetin) and 275.14, respectively. Ellagic acid (RT: 19.71 min, *m*/*z* 301.08) exhibited a high-intensity base peak with fragments at *m*/*z* 285.11, 217.16, and 143.18. The ion at *m*/*z* 285.11 corresponds to the loss of a hydroxyl radical (–*OH*, 17 Da), a common initial fragmentation of polyphenols due to the lability of the phenolic OH group. The fragment at *m*/*z* 217.16 was attributed to retro-Diels–Alder (RDA) cleavage within one of the lactone rings, a well-documented mechanism in the degradation of ellagitannins and related phenolic scaffolds. The *m*/*z* 143.19 ion likely results from extensive ring opening and cleavage, producing a stabilized phenolic moiety. These fragment ions are consistent with literature reports of ellagic acid fragmentation behavior in negative ESI-MS/MS, supporting its structural assignment and chemotaxonomic relevance in polyphenol-rich botanical extracts.

Sesquiterpenoid features were confirmed by isozonarol (RT: 5.57 min, *m*/*z* 313.04), which displayed a complex fragmentation pattern with prominent ions at *m*/*z* 151.14 and 294.62. The fragment at *m*/*z* 294.62 corresponds to the loss of a water molecule (−18 Da), which is indicative of a free hydroxyl group in the structure, which is typical of sesquiterpene phenols. The most prominent diagnostic fragment at *m*/*z* 151.14 suggests retro-ene cleavage or aromatic ring scission, producing a stable phenoxide-containing substructure. This ion reflects the core hydroquinone or chromene moiety, supporting the partial degradation of the drimane-like structure of the compound. These fragmentation pathways confirm the meroterpenoid nature of isozonarol and support its tentative identification as a chemotaxonomic marker in sesquiterpenoid-rich plants.

#### 2.2.2. LC/MS/MS of Lemon Juice (L) Extract

Lemon extracts predominantly contain flavanones, flavones, organic acids, and monoterpenoids (Table 2, Supplementary Table S3). Citric acid (RT: 2.41 min, *m*/*z* 174.94) fragmented into *m*/*z* 111.01 and 129.04, in accordance with the expected cleavage of carboxyl functionalities. Citric acid (C_6_H_8_O_7_), a tricarboxylic acid widely distributed in plant tissues, was detected as a deprotonated molecular ion at *m*/*z* 174.94 in the negative ESI mode, closely matching its theoretical [M–H_2_O]^−^ mass (MW = 192.027 g/mol).

The MS/MS spectrum revealed a complex fragmentation pattern indicative of its polyacidic nature, with prominent fragment ions observed at *m*/*z* 159.02, 146.99, 138.97, 118.96, and 110.95. The fragment at *m*/*z* 146.99 may arise from the concurrent loss of water and carbon monoxide (–*H*_2_*O*–*CO*, 45 Da), which is indicative of decarbonylation and dehydration. Ions at *m*/*z* 138.97 and 118.96 were consistent with sequential neutral losses, including further decarboxylation (–*COOH*, 46 Da), and internal rearrangement processes. The *m*/*z* 110.95 ion represents a deeper cleavage within the citric acid skeleton, yielding a stable anionic fragment from the central carbon framework. Citric acid enhances extraction efficiency and contributes to metal chelation and pH-dependent molecular release [42].

Flavanones, such as hesperidin (RT: 9.33 min, *m*/*z* 609.11), produced typical product ions at *m*/*z* 301.07 (hesperetin) and *m*/*z* 325.04, whereas eriocitrin (RT: 8.16 min, *m*/*z* 594.94) yielded a major aglycone peak at *m*/*z* 287.06 (eriodictyol). Narirutin (RT: 8.90 min, *m*/*z* 578.95) and diosmetin (RT: 10.82 min, *m*/*z* 299.05). Kaempferol (RT: 13.20 min, *m*/*z* 285.04) exhibited classic flavonol fragmentation to *m*/*z* 151 and 133 via Retro-Diels–Alder (RDA) reactions. Monoterpenes, such as citral (RT: 3.81 min, *m*/*z* 151.04) and linalool (RT: 4.60 min, *m*/*z* 152.98), were identified based on their low molecular weights and characteristic isoprenoid fragment losses. The bitter limonoid, limonin (RT: 13.85 min, *m*/*z* 469.03), produced ions at *m*/*z* 229.18 and 381.13, which is in agreement with the reported fragmentation pattern of limonoid lactones. Citrus-specific flavonoids, including eriocitrin, narirutin, diosmetin, and hesperidin, were detected in the lemon extracts. These compounds modulate oxidative stress and neuroinflammation, with recent studies demonstrating the ability of hesperidin to enhance synaptic plasticity via the Wnt/β-catenin pathway [43]. Monoterpenes, such as linalool and citral, contribute to the neuroprotective and aromatic properties of this extract. In addition, limonoids, such as limonin, which was uniquely detected in lemon, have been shown to have neuroprotective activity, supporting their anti-Alzheimer’s disease activity [44,45].

#### 2.2.3. LC/MS/MS of Lemon Juice-Assisted Strawberry Extract (S/L) Extract

The combined S/L extract exhibited an expanded metabolite profile, with 24 tentatively identified compounds, many of which were either absent or present in the individual extracts (Table 3, Supplementary File Table S4). Daphnetin (RT: 1.10 min, *m*/*z* 177.00) displayed a fragmentation pattern consistent with a coumarin backbone, producing fragments at *m*/*z* 129.02 and 148.95. Tormentic acid (RT: 7.40 min, *m*/*z* 487.11) yielded multiple fragments, including *m*/*z* 374.54 and 424.63, consistent with triterpenoid fragmentation via the sequential loss of hydroxyl and carboxyl moieties.

Peonidin-3-glucoside (RT: 23.00 min, *m*/*z* 461.25) and pelargonidin-3-malonylglucoside (RT: 24.43 min, *m*/*z* 498.07) fragmented into aglycone ions at *m*/*z* 279.22 and 270.07, respectively, confirming the presence of O-glycosidic anthocyanins. In addition, metabolites such as thymidine (RT: 9.06 min, *m*/*z* 241.07) showed diagnostic ions at *m*/*z* 194.99 and 154.97, confirming the presence of nucleoside structures through cleavage between deoxyribose and the base.

Chrysin (RT: 20.94 min, *m*/*z* 253.04) produced fragments at *m*/*z* 152.08 and 138.06, resulting from RDA ring scission, which is typical of flavones. Other unique constituents included 2,4-bis(1,1-dimethylethyl)-phenol (RT: 22.98 min, *m*/*z* 205.11) and benzaldehyde derivatives, further supporting the enhanced complexity of the S/L profile of the essential oil. The presence of organic acids, such as 2-oxobutyric acid (RT: 7.85 min, *m*/*z* 100.95), and sugar alcohols, such as threitol (RT: 4.78 min, *m*/*z* 121.05), indicates the preservation of the primary metabolites.

Tormentic acid (a triterpene), chrysin (a flavonoid), and daphnetin (a coumarin) were absent from both individual fruit extracts but liberated during acid-mediated co-extraction. Cui et al. [46] demonstrated that tormentic acid significantly improved memory and reduced neuroinflammation in a mouse model of Alzheimer’s disease. This compound enhanced cognitive function and suppressed pro-inflammatory cytokines in the hippocampus, primarily by inhibiting the *NF-κB* signaling pathway. Chrysin preserves dopaminergic neurons, enhances cognitive performance, and improves motor function, supporting its therapeutic potential in neurological disorders such as Parkinson’s and Alzheimer’s diseases [47,48]. Moreover, Daphnetin exhibits significant neuroprotective effects, as shown by Zhi et al. [49] and Qi et al. [50]. It protects hippocampal neurons from oxygen-glucose deprivation and oxidative stress by inhibiting apoptosis and modulating key pathways, such as MAPK signaling and HSP70 expression. These findings highlight the potential of daphnetin to prevent neuronal damage and promote neuronal survival under stressful conditions.

Key molecules, such as gallic acid, chlorogenic acid, ellagic acid, and peonidin-3-glucoside, were identified in the three extracts (S, L, and S/L), indicating that the acidic nature of the lemon matrix not only preserves these important bioactives but also aids in the development of new therapeutic agents. This preservation of polyphenols aligns with previous findings that acidic environments can stabilize anthocyanins and phenolic acids, thereby maintaining their bioactivity under co-extraction conditions [42,51]. Additionally, the presence of carbohydrate derivatives (turanose and threitol), aldehydes (benzaldehyde and its methoxy derivative), and low-molecular-weight organic acids suggests that acid-catalyzed hydrolysis, esterification, or Maillard-type reactions may have occurred during extraction. These chemical transformations are known to alter the solubility, enhance the antioxidant activity, and modulate the bioavailability in plant-based systems [52].

### 2.3. Biological Activity of Extracts (S, L, S/L)

#### 2.3.1. Behavioral Assessment of Extracts in AlCl_3_-Induced Alzheimer’s Model

Behavioral tests were conducted to evaluate the neuroprotective efficacy of strawberry and/or lemon extracts in ameliorating AlCl_3_-induced cognitive impairment, particularly in learning and memory functions.

#### 2.3.2. Morris Water Maze (MWM) Test

**Figure 3:**
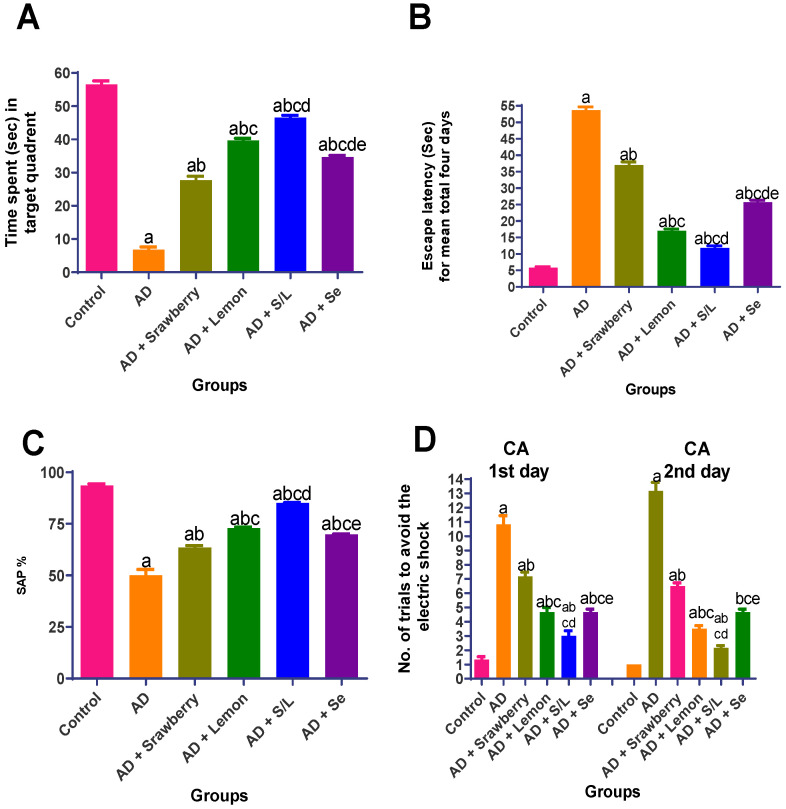
Effect of Strawberry and/or Lemon on Behavioral Tests in AlCl_3_-induced AD: (**A**) Time spent in the target quadrant in the MWM test. (**B**) Escape latency over four days in the MWM test. (**C**) SAP (%) in the Y-maze test. (**D**) Number of trials to avoid electric shock in the CA test. Data are presented as mean ± SE (*n* = 6). Significance (a): relative to the control group; (b): relative to the AD group; (c): relative to AD + Strawberry; (d): relative to AD + Lemon; and (e): relative to AD+ S/L. Significance: *p* < 0.05. AD: Alzheimer’s disease; S/L: a Combination of Strawberry and Lemon; CA: Conditioned avoidance; MWM: Morris water maze testing; and SAP: Spontaneous alternation percentage.

In the MWM paradigm, AlCl_3_-intoxicated rats exhibited a pronounced decline in spatial learning and memory, as evidenced by a significant (*p* < 0.05) reduction in the time spent in the target quadrant (88% decrease) and a marked increase in escape latency (9.2-fold) over the four-day training period compared to the control group (Figure 3A,B). Treatment with strawberry extract or selenium (Se) partially reversed these deficits, increasing the time spent in the target quadrant by 4.1- and 5.1-fold, respectively, and reducing escape latency by 31.1% and 52%, respectively, compared to the AlCl_3_ group. Lemon treatment yielded a more pronounced improvement, reflected by a 5.8-fold increase in the target quadrant time and a 68.3% reduction in escape latency. Remarkably, co-administration of strawberry and lemon extracts demonstrated the most substantial protective effect, resulting in a 6.8-fold increase in residence time within the target quadrant and a 78% decrease in escape latency compared with the AlCl_3_-treated group.

#### 2.3.3. Y-Maze Spontaneous Alternation Test

AlCl_3_ exposure significantly impaired spatial working memory, as indicated by a 46.5% reduction in spontaneous alternation performance (SAP%) relative to that of the control group (Figure 3C). Administration of strawberry extract and selenium led to notable improvements, increasing SAP% by approximately 27% and 39.7%, respectively, compared to the AlCl_3_ group. Lemon treatment further enhanced cognitive performance, restoring the SAP% by 45.7%. The combination of strawberry and lemon extracts exerted the most robust effect, resulting in a 70% increase in SAP%, thereby indicating a synergistic enhancement in spatial working memory in AlCl_3_-induced AD rats.

#### 2.3.4. Conditioned Avoidance Test (CA)

As illustrated in Figure 3D, rats in the AlCl_3_-induced AD group exhibited substantial impairment in avoidance learning, as demonstrated by an 8.1-fold and 13.2-fold increase in the number of trials required to avoid the electric shock on the first and second days, respectively, compared with the control group. Treatment with strawberry extract significantly reduced the number of avoidance trials by approximately 33.8% on day one and 50.7% on day two relative to the AD group. The selenium-treated group showed a more pronounced effect, with reductions of 56.9% and 64.6% on days one and two, respectively. Similarly, lemon extract markedly decreased the number of avoidance trials by 56.9% on the first day and 73.4% on the second day. Notably, the combination of strawberry and lemon extracts yielded the greatest improvement, reducing the required avoidance trials by 72.3% and 83.6% on the first and second days, respectively, compared to the AD group (*p* < 0.05), indicating a robust enhancement in associative learning and memory retention.

### 2.4. Effect of Extracts on Oxidative Stress in Brain Tissues

**Figure 4:**
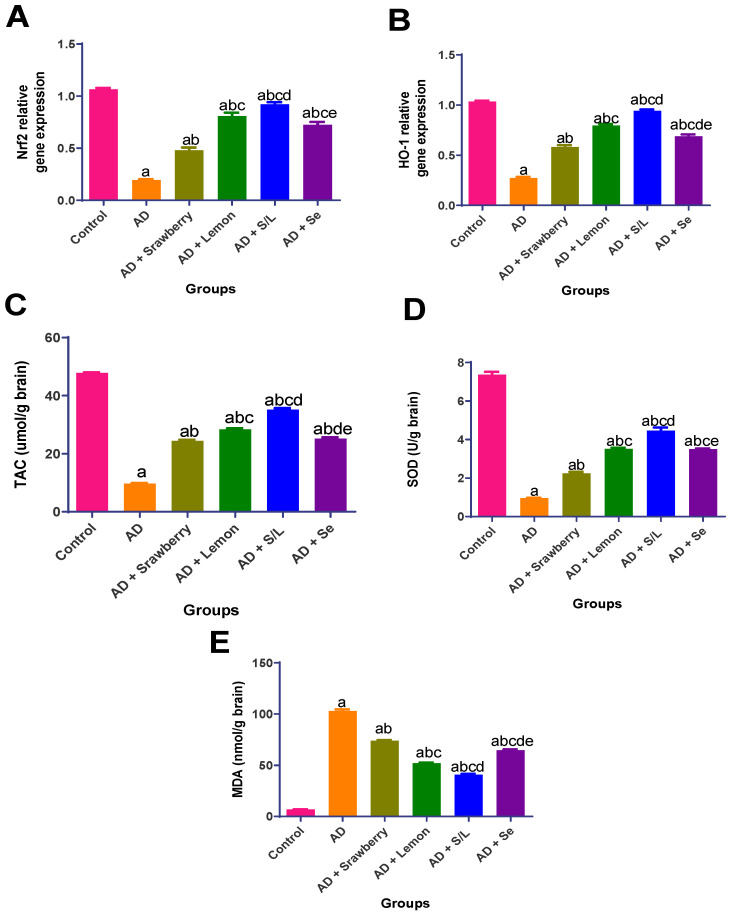
Effect of Strawberry and/or lemon on oxidative stress in AlCl_3_-induced AD: (**A**) *Nrf2* (**B**) *HO-1* (**C**) TAC (**D**) SOD (**E**) MDA. Data are presented as mean ± SE (*n* = 6). Significance (a): relative to the control group; (b): relative to the AD group; (c): relative to AD + Strawberry; (d): relative to AD + Lemon; and (e): relative to AD + S/L. Significance: *p* < 0.05. AD: Alzheimer’s disease; S/L: a Combination of Strawberry and Lemon; *Nrf2*: Nuclear factor erythroid 2-related factor 2; *HO-1*: Hemoxygenase-1; TAC: total antioxidant capacity; SOD: superoxide dismutase; and MDA: malondialdehyde.

As shown in Figure 4, AlCl _3_ administration induced pronounced oxidative stress, as evidenced by a significant reduction in the levels of key endogenous antioxidants. Specifically, nuclear factor erythroid 2–related factor 2 (*Nrf2*), heme oxygenase-1 (*HO-1*), total antioxidant capacity (TAC), and superoxide dismutase (SOD) activity decreased by 81.7%, 73.6%, 79.7%, and 86.9%, respectively, compared to the control group (Figure 4A–D). Additionally, AlCl_3_ exposure led to (15.1-fold) increase in brain malondialdehyde (MDA) levels, indicating enhanced lipid peroxidation (Figure 4E). In contrast, treatment with strawberry extract significantly restored antioxidant defenses, resulting in (2.5-, 2.1-, 2.4-, and 2.3-fold) increases in *Nrf2*, *HO-1*, TAC, and SOD activity, respectively, along with a 27.9% reduction in MDA levels, relative to the AD group. Selenium treatment demonstrated greater efficacy, elevating *Nrf2*, *HO-1*, TAC, and SOD levels (3.7-, 2.5-, 2.6-, and 3.6-fold, respectively) and decreasing MDA content by 37%. Lemon treatment yielded even more pronounced effects, increasing *Nrf2*, *HO-1*, TAC, and SOD levels (4.2-, 2.8-, 2.9-, and 3.6-fold, respectively) and reducing MDA levels by 49.3%. Notably, co-administration of strawberry and lemon extracts produced the most substantial antioxidant response, with *Nrf2*, *HO-1*, TAC, and SOD levels elevated (4.7-, 3.5-, 3.6-, and 4.6-fold, respectively), and MDA levels reduced by 60.2% compared to the AD group, indicating a synergistic protective effect against AlCl_3_-induced oxidative damage.

### 2.5. Effect of Extracts on Brain Neurotransmitters Levels

**Figure 5:**
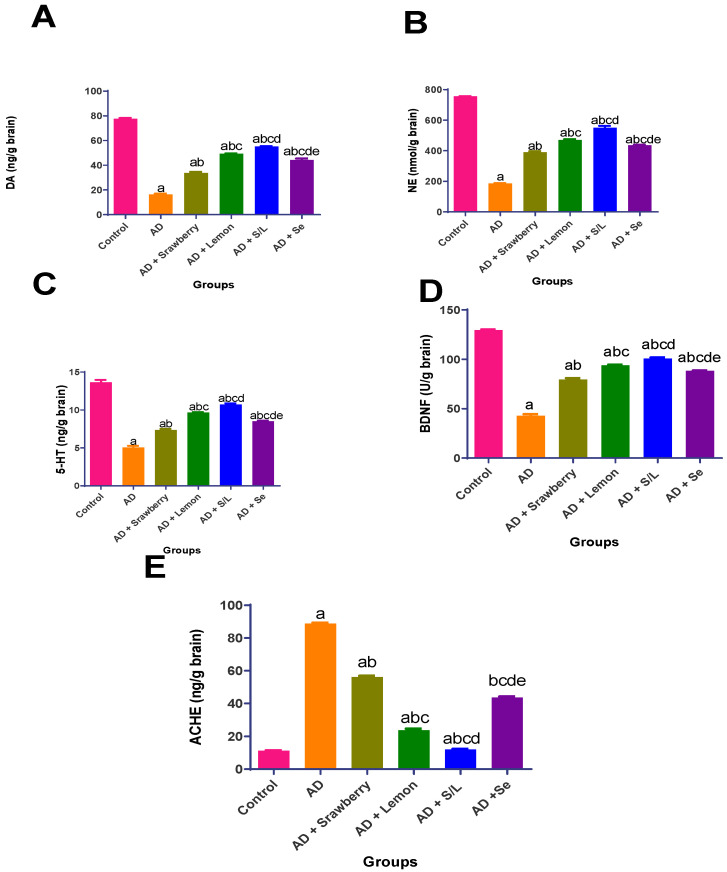
Effect of Strawberry and/or lemon on brain neurotransmitters in AlCl_3_-induced AD: (**A**) DA (**B**) NE (**C**) 5-HT (**D**) ACHE (**E**) BDNF. Data are presented as mean ± SE (*n* = 6). Significance (a): relative to the control group; (b): relative to the AD group; (c): relative to AD + Strawberry; (d): relative to AD + Lemon; and (e): relative to AD+ S/L. Significance: *p* < 0.05. AD, Alzheimer’s disease; S/L: a Combination of Strawberry and Lemon; DA, dopamine; NE, norepinephrine; 5-HT, serotonin; ACHE, acetylcholinesterase; BDNF, brain-derived neurotrophic factor.

As illustrated in Figure 5, the Alzheimer’s disease (AD) group demonstrated a significant decline in the levels of key neurotransmitters and neurotrophic factors. Specifically, dopamine (DA), norepinephrine (NE), serotonin (5-HT), and brain-derived neurotrophic factor (BDNF) levels were reduced by 78.8%, 75.5%, 64.9%, and 62.9%, respectively, compared to those in the control group. In contrast, strawberry treatment markedly improved neurotransmitter levels, resulting in 2.1-fold increases in both DA and NE, a 1.5-fold increase in 5-HT, a 1.9-fold increase in BDNF, and a 36.8% elevation in acetylcholinesterase (AChE) levels relative to the AD group. Selenium (Se) administration produced a greater neuroprotective effect, with 2.7-, 2.4-, 1.7-, and 2.1-fold increases in DA, NE, 5-HT, and BDNF levels, respectively, accompanied by a 50.9% reduction in AChE levels. Lemon treatment further enhanced these effects, yielding 3-, 2.5-, 1.9-, and 2.2-fold increases in DA, NE, 5-HT, and BDNF, respectively, and elevating AChE levels by 73.1% compared to the AD group. Notably, the combined administration of strawberry and lemon extracts elicited the most significant response, with increases of 3.4-, 3-, 2.1-, and 2.4-fold in the respective neurotransmitter levels, along with a substantial 86.4% reduction in AChE activity, indicating a synergistic restoration of the neurochemical balance in AD rats.

### 2.6. Effect of Extracts on the Neuroinflammatory Biomarkers

**Figure 6:**
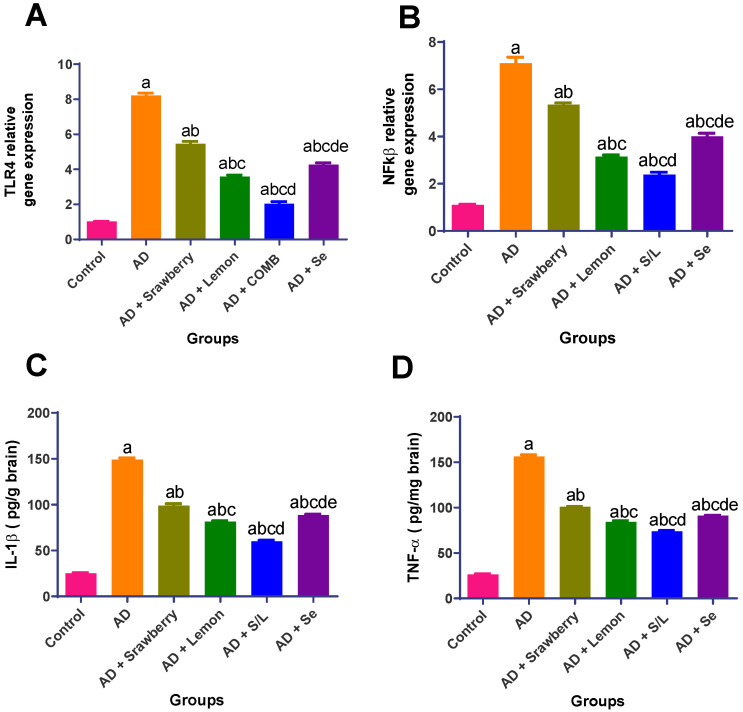
Effect of Strawberry and/or Lemon on Neuroinflammatory Biomarkers in AlCl_3_-induced AD: (**A**) *TLR4* (**B**) NFκβ (**C**) 5-IL-1β (**D**) TNF-α. Data are presented as mean ± SE (*n* = 6). Significance (a): relative to the control group; (b): relative to the AD group; (c): relative to AD + Strawberry; (d): relative to AD + Lemon; and (e): relative to AD+ S/L. Significance: *p* < 0.05. AD: Alzheimer’s disease; S/L: a Combination of Strawberry and Lemon; TLR-4: Toll-like receptor-4; NFκB: Nuclear factor kappa B; IL-1β: interleukin-1β; TNF-α: tumor necrosis factor-alpha.

The mRNA expression levels of the pro-inflammatory markers toll-like receptor 4 (*TLR4*) and nuclear factor-kappa B (*NF-κB*) were significantly upregulated by 8.1-fold and 6.4-fold, respectively, in AlCl_3_-induced AD rats compared to those in the control group. This was accompanied by a marked elevation in the protein levels of interleukin-1β (IL-1β) and tumor necrosis factor-α (TNF-α), both increased by 5.9-fold. Strawberry administration attenuated the AlCl_3_-induced inflammatory response, as reflected by reductions in *TLR4* and *NF-κB* expression by 33.5% and 24.7%, respectively, alongside significant decreases in IL-1β and TNF-α levels by 33.5% and 35.4%, respectively, relative to the AD group. Selenium treatment yielded a greater anti-inflammatory effect, downregulating *TLR4* and *NF-κB* expression by 48.1% and 43.7%, respectively, and reducing IL-1β and TNF-α levels by 40.5% and 41.6%, respectively. Lemon treatment demonstrated superior efficacy in mitigating AlCl_3_-induced neuroinflammation, with reductions in *TLR4* and *NF-κB* expression of 56.4% and 55.6%, respectively, and corresponding decreases in IL-1β and TNF-α levels of 45.4% and 45.9%, respectively. Notably, the co-administration of strawberry and lemon extracts exhibited the most pronounced anti-inflammatory activity, resulting in the downregulation of *TLR4* and *NF-κB* by 75.3% and 66.4%, and suppression of IL-1β and TNF-α levels by 59.8% and 52.7%, respectively, compared to the AD group (Figure 6).

### 2.7. Effect of Extracts on Pathophysiology and Inflammasome Activation Biomarkers

**Figure 7:**
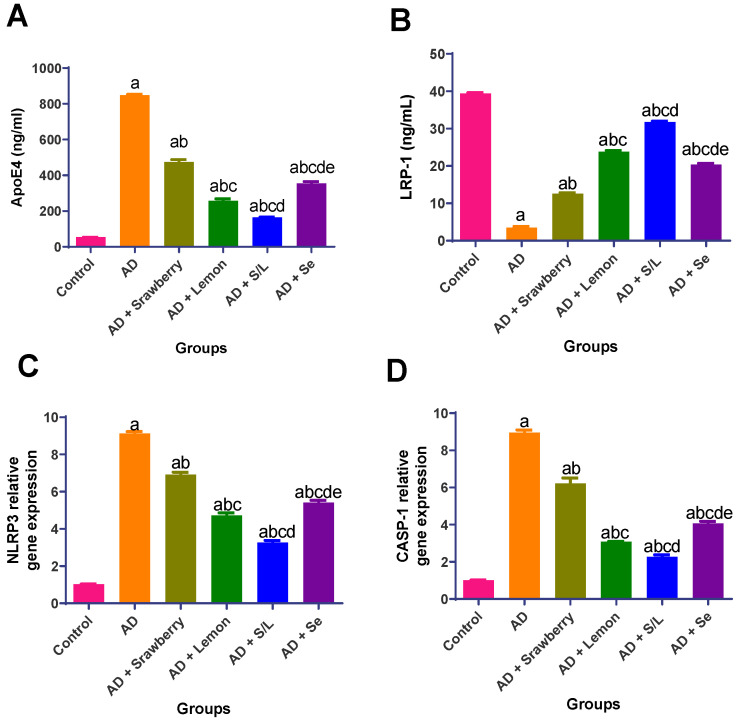
Effect of Strawberry and/or Lemon on AD pathophysiology and Inflammasome Activation Biomarkers in AlCl_3_-induced AD: (**A**) ApoE4 (**B**) LRP-1 (**C**) *NLRP3* (**D**) *CASP-1*. Data are presented as mean ± SE (*n* = 6). Significance (a): relative to the control group; (b): relative to the AD group; (c): relative to AD + Strawberry; (d): relative to AD + Lemon; and (e): relative to AD+ S/L. Significance: *p* < 0.05. AD, Alzheimer’s disease; S/L: a Combination of Strawberry and Lemon; ApoE4: Apolipoprotein E variant 4; LRP1: Low-density lipoprotein receptor-related protein-1; *NLRP3*, ACHT, LRR, and PYD domains-containing protein 3; *CASP-1*: Caspase-1.

As shown in Figure 7, AlCl_3_ administration resulted in pronounced dysregulation of inflammasome-related markers and lipid metabolism in the kidney. Specifically, a substantial reduction in low-density lipoprotein receptor-related protein 1 (LRP1) expression was observed (11.3-fold decrease), alongside significant elevations in apolipoprotein E4 (ApoE4), nucleotide-binding domain leucine-rich repeat and pyrin domain-containing protein 3 (*NLRP3*), and caspase-1 (*CASP-1*) expression levels by 15.9-, 8.8-, and 8.8-fold, respectively, compared to the control group. In the AD + Strawberry group, LRP1 expression increased 3.6-fold, while ApoE4, *NLRP3*, and *CASP-1* levels were reduced by 44%, 24.1%, and 30.5%, respectively, compared to the AD group. Similarly, selenium treatment elevated LRP1 expression by 5.8-fold and decreased ApoE4, *NLRP3*, and *CASP-1* levels by 58%, 40.6%, and 54.6%, respectively. Lemon administration also yielded significant improvements in AD pathophysiology, increasing LRP1 expression by 6.8-fold and reducing ApoE4, *NLRP3*, and *CASP-1* levels by 69.6%, 48.2%, and 65.6%, respectively, compared to the AD group. The combined administration of strawberry and lemon extracts produced the most profound therapeutic effect, exhibiting the greatest restoration of LRP1 expression (9.1-fold increase) and the most substantial reductions in ApoE4, *NLRP3*, and *CASP-1* expression levels by 80.6%, 64.2%, and 74.6%, respectively, compared to the AD group, surpassing the effects of either treatment alone.

### 2.8. Effect of Extracts on Apoptosis Biomarkers

**Figure 8:**
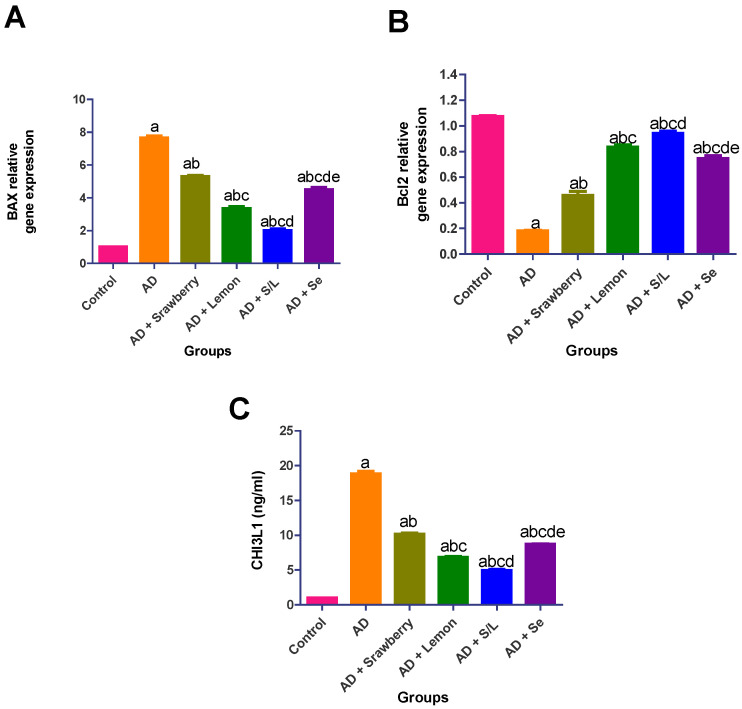
Effect of Strawberry and/or lemon on apoptosis in AlCl_3_-induced AD: (**A**) *BAX* (**B**) Bcl2 (**C**) CHI3L. Data are presented as mean ± SE (*n* = 6). Significance (a): relative to the control group; (b): relative to the AD group; (c): relative to AD + Strawberry; (d): relative to AD + Lemon; and (e): relative to AD+ S/L. Significance: *p* < 0.05. AD: Alzheimer’s disease; S/L: strawberry and lemon combination; *BAX*: bcl-2-like protein 4; Bcl2: B-cell lymphoma 2; CHI3L: Chitinase-3-like protein 1.

As shown in Figure 8, AlCl_3_-intoxicated rats exhibited substantial dysregulation of apoptosis-related markers and tissue injury markers. Specifically, *BAX* gene expression was upregulated by approximately 7.6-fold, whereas *Bcl-2* gene expression was downregulated by 5.9-fold. Additionally, the levels of the tissue injury biomarker chitinase-3-like protein 1 (*CHI3L1*) were elevated by 18.2-fold compared to those in the control group. Strawberry extract administration significantly mitigated these effects, reducing *BAX* expression by 30.5%, increasing *Bcl-2* expression by 2.5-fold, and decreasing *CHI3L1* levels by 46.1% relative to the AD group. Selenium treatment further improved these parameters, downregulating *BAX* and *CHI3L1* expression by 41% and 53.6%, respectively, and upregulating *Bcl-2* expression by 4.1-fold compared to AD rats. Lemon juice extract exerted a more pronounced anti-apoptotic and tissue-protective effect, evidenced by a 56.2% and 63.6% reduction in *BAX* and *CHI3L1* levels, respectively, and a 4.6-fold increase in *Bcl-2* expression. The most significant restoration was observed in the group receiving the combined treatment of strawberry and lemon, which achieved a 73.4% and 73.8% reduction in *BAX* and *CHI3L1* expression, respectively, and a 5.2-fold upregulation of *Bcl-2*, indicating robust neuroprotection against AlCl_3_-induced apoptosis and tissue injury.

### 2.9. Effect of Extracts on Potential AD Biomarkers (BACE1, APP, p-Tau, and Aβ)

**Figure 9:**
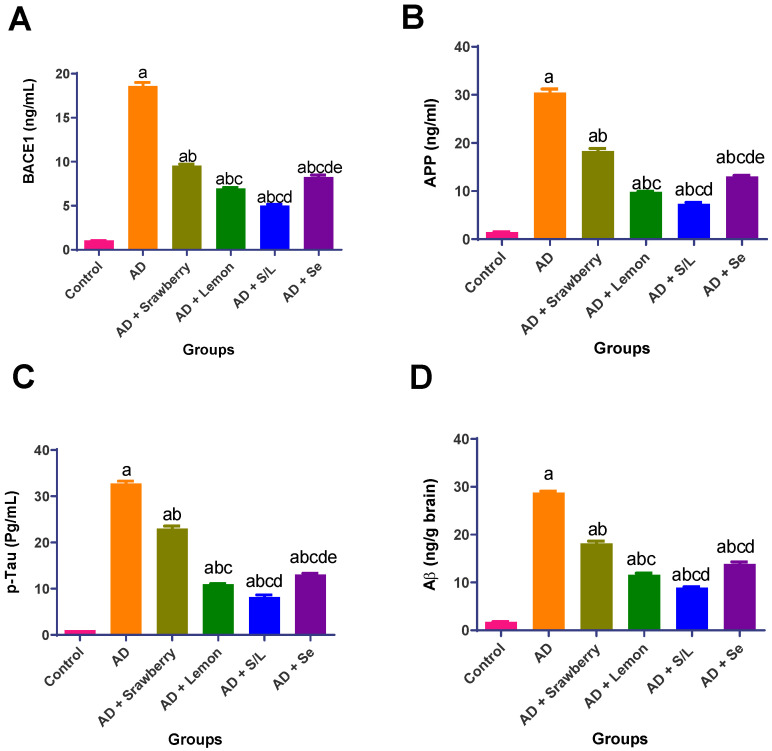
Effect of Strawberry and/or Lemon on AD biomarkers in AlCl_3_-induced AD: (**A**) BACE1 (**B**) APP (**C**) *p*-Tau (**D**) Aβ. Data are presented as mean ± SE (*n* = 6). Significance (a): relative to the control group; (b): relative to the AD group; (c): relative to AD + Strawberry; (d): relative to AD + Lemon; and (e): relative to AD+ S/L. Significance: *p* < 0.05. AD, Alzheimer’s disease; S/L: a Combination of Strawberry and Lemon; BACE1: Beta-site amyloid precursor protein cleaving enzyme 1 (β-secretase); APP: Amyloid precursor protein; *p*-tau, phosphorylated tau; and Aβ: Amyloid beta.

As shown in Figure 9, AlCl_3_ administration induced significant pathological alterations associated with Alzheimer’s disease, evidenced by marked upregulation of β-site amyloid precursor protein cleaving enzyme 1 (BACE1), amyloid precursor protein (APP), and phosphorylated tau (*p*-Tau) by 17.7-, 20.5-, and 32.1-fold, respectively, compared to the control group (*p* < 0.05). This was accompanied by a 16.4-fold increase in amyloid-β (Aβ) levels, confirming the enhanced amyloidogenic processing. Strawberry extract treatment significantly attenuated Aβ accumulation and tau hyperphosphorylation, reducing BACE1, APP, *p*-Tau, and Aβ levels by 48.5%, 39.9%, 29.9%, and 36.9%, respectively, relative to the AD group (*p* < 0.05). Similarly, selenium administration markedly suppressed these AD biomarkers by 55.6%, 57.2%, 60.1%, and 51.8%. Lemon extract exerted an even stronger neuroprotective effect, significantly lowering the levels of BACE1, APP, *p*-Tau, and Aβ by 62.6%, 66.5%, 59.7%, and 67.6%, respectively, compared to those in the AD group. Combined administration of strawberry and lemon produced the most robust therapeutic response, reducing BACE1, APP, *p*-Tau, and Aβ levels by 73.1%, 75.9%, 74.9%, and 69.0%, respectively, relative to the AD group, indicating a synergistic effect in mitigating amyloidogenic and tau-related pathologies.

### 2.10. Effect of Extracts on Wnt3/β-Catenin/GSK3β Signaling Pathway

**Figure 10:**
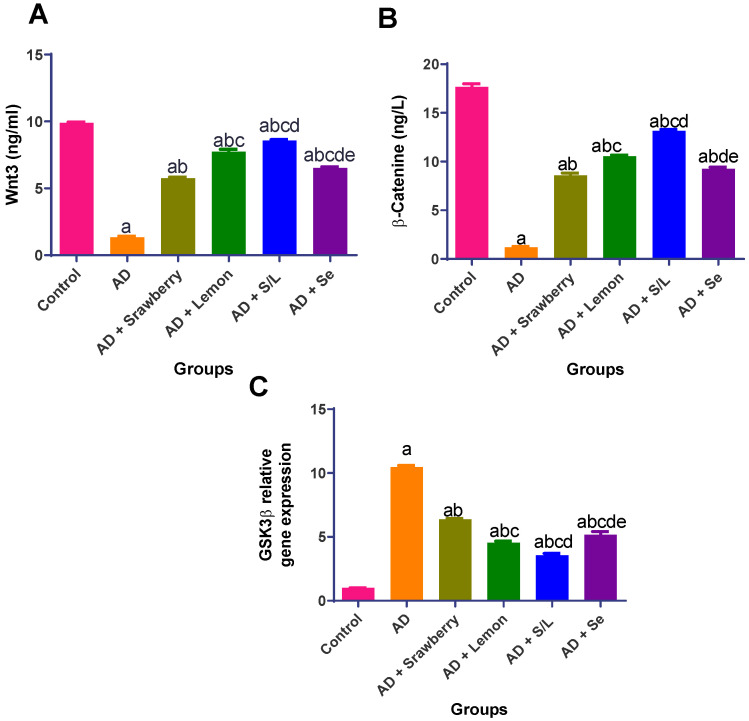
Effect of Strawberry and/or Lemon on Wnt3/β-Catenin/*GSK3β* signaling pathway in AlCl_3_-induced AD: (**A**) Wnt3 (**B**) β-catenin (**C**) *GSK3β*. Data are presented as mean ± SE (*n* = 6). Significance (a): relative to the control group; (b): relative to the AD group; (c): relative to AD + Strawberry; (d): relative to AD + Lemon; and (e): relative to AD+ S/L. Significance: *p* < 0.05. AD, Alzheimer’s disease; S/L: a Combination of Strawberry and Lemon; *GSK3β*: Glycogen synthase kinase-3β.

As shown in Figure 10, AlCl_3_ administration resulted in a pronounced disruption of the Wnt/β-catenin signaling pathway, as evidenced by a significant reduction in Wnt3 and β-catenin levels by 7.4- and 14.8-fold, respectively, along with a 10.3-fold upregulation of glycogen synthase kinase-3β (*GSK3β*) gene expression compared to the control group (*p* < 0.05). These alterations were notably reversed by strawberry treatment, which elevated Wnt3 and β-catenin levels by 4.3- and 7.2-fold, respectively, while downregulating *GSK3β* expression by 39.1% relative to the AD group (*p* < 0.05). Similarly, selenium treatment resulted in 4.9- and 7.8-fold increases in Wnt3 and β-catenin, respectively, accompanied by a 50.5% reduction in *GSK3β* expression compared with the AD group. Lemon administration showed an even greater effect in restoring Wnt3/β-catenin/*GSK3β* signaling, significantly increasing Wnt3 and β-catenin levels by 5.8- and 8.8-fold, respectively, and suppressing *GSK3β* expression by 56.5% compared to AlCl_3_-treated rats. The combination therapy of strawberry and lemon demonstrated the most robust restoration of Wnt/β-catenin signaling, increasing Wnt3 and β-catenin levels by 6.4- and 11-fold, respectively, and reducing *GSK3β* expression by 66% compared with that in the AD group. In the current study, this combination exhibited superior efficacy to selenium, indicating enhanced neuroprotective potential compared to the standard reference treatment for Alzheimer’s disease.

### 2.11. Histopathological Evaluation of Brain Tissues

**Figure 11:**
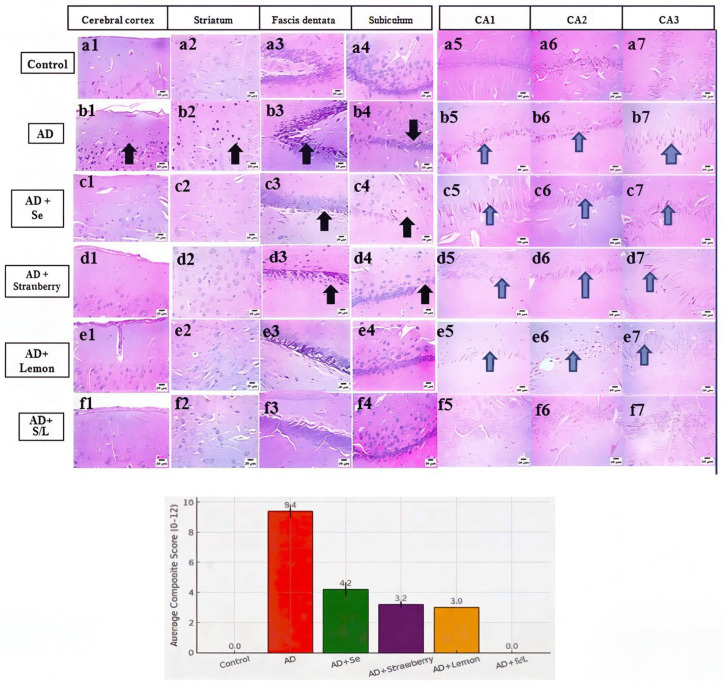
Effect of Strawberry and/or Lemon on AlCl_3_-induced histopathological alterations. (**a1**–**f7**) Photomicrographs representing staining of brain sections (cerebral cortex, striatum, fascia dentata, subiculum, CA1, CA2 and CA3) with H & E (scale bar 25 μm). (**a1**–**a7**)) Control group, (**b1**–**b7**) AD group, (**c1**–**c7**) AD + Se-treated group, (**d1**–**d7**) AD + Strawberry-treated group, (**e1**–**e7**) AD + Lemon-treated group, and (**f1**–**f4**) AD + S/L-treated group. In the brain sections, the black arrow indicates nuclear pyknosis and degeneration. Meanwhile, blue arrow indicates necrotic neurons. AD, Alzheimer’s disease; Se, Selenium; S/L, a combination of strawberry and lemon. Average composite non-intact neuron damage scores (mean ± SEM) for each experimental group. Higher scores indicate greater overall neuronal damage (maximum possible score = 12, corresponding to severe damage in all four regions). The untreated AD group showed the highest average composite score (~9.4, red bar), reflecting widespread severe neuronal damage. In contrast, the control and AD + S/L combined treatment groups both exhibited average scores of ~0 (no damage). Intermediate treatments (AD + Se, AD + Strawberry, and AD + Lemon) showed progressively lower composite scores than untreated AD, indicating partial protection against neuronal damage.

Histopathological examination of control brain tissues revealed a normal neuronal architecture in the cerebral cortex, striatum, fascia dentata, and subiculum (Figure 11(a1–a4)). In contrast, AlCl_3_-treated (AD) rats exhibited marked neurodegeneration, characterized by shrunken, degenerated neurons and pronounced nuclear pyknosis in the cerebral cortex and striatum, as well as severe nuclear pyknosis in the fascia dentata and subiculum (black arrows) (Figure 11(b1–b4)). Selenium treatment partially restored neuronal integrity, displaying a normal histological appearance in the cerebral cortex and striatum, mild nuclear pyknosis in the fascia dentata, and moderate nuclear pyknosis in the subiculum (Figure 11(c1–c4)). Similarly, strawberry treatment preserved the normal structure of neurons in the cerebral cortex and striatum, with moderate nuclear pyknosis in the fascia dentata and mild pyknosis in the subiculum (Figure 11(d1–d4)). Lemon administration resulted in broader histological improvement, preserving normal neuronal architecture in the cerebral cortex, striatum, and subiculum, while only moderate nuclear pyknosis was observed in the fascia dentata (Figure 11(e1–e4)). Notably, combined treatment with strawberry and lemon extracts resulted in complete histological preservation, with normal neuronal morphology evident across all examined regions, including the cerebral cortex, striatum, fascia dentata, and subiculum (Figure 11(f1–f4)).

Quantitative scoring of skin healing supported these histological observations. The control group exhibited consistently low neuron counts across all regions, resulting in a composite score of 0.0 ± 0.0, indicating intact neuronal architecture. In contrast, the AD group displayed markedly elevated neuronal damage, with a mean composite score of 9.4 ± 0.4. This reflects extensive and consistent damage across multiple brain regions, particularly the frontal cortex, which has the highest individual region scores. Selenium administration (AD + Se) significantly attenuated neurodegeneration, yielding a reduced composite score of 4.2 ± 0.5. The protective effect was most notable in the corpus callosum and striatum, although some samples exhibited severe damage in the FD region. Similarly, strawberry supplementation (AD + Strawberry) further improved neuronal integrity, with a mean score of 3.2 ± 0.2, while lemon-treated animals (AD + Lemon) demonstrated a consistent composite score of 3.0 ± 0.0, with damage restricted predominantly to the FD region. Remarkably, co-administration of strawberry and lemon extracts (AD + S/L) completely preserved neuronal integrity, as evidenced by a composite score of 0.0 ± 0.0 for all samples. None of the animals in this group displayed non-intact neuron counts exceeding five in any evaluated region. A graphical comparison of the average composite scores across groups is presented in.

## 3. Discussion

Alzheimer’s disease (AD) is characterized by progressive neurodegeneration involving oxidative stress, neuroinflammation, mitochondrial dysfunction, and amyloid beta (Aβ) accumulation [35]. Strawberries are rich in bioactive nutrients, and research has demonstrated their potential to mitigate oxidative stress and reduce DNA damage [53]. Considering the lack of effective treatments for age-related cognitive decline, a cost-benefit analysis indicates that the integration of safe foods offers a positive value proposition. Diets rich in anthocyanins and flavonoids have been associated with a reduced risk of developing Alzheimer’s disease and related dementia [54]. The consumption of foods with a higher long-term intake of flavonoids, such as berry fruits, may reduce the risk of Alzheimer’s disease and related dementias [55]. Berry fruits, rich in polyphenolic compounds with anti-inflammatory and antioxidant properties, present a viable approach to counteract the adverse effects of chronic oxidative stress and inflammation. These conditions are associated with pathological brain aging [37].

The analysis of strawberry (S), selenium (Se), lemon (L), and their combination (S/L) in the AlCl_3_-induced Alzheimer’s model demonstrated that each intervention exerted measurable neuroprotective effects, as reflected by consistent improvements in behavioral, biochemical, molecular, and histological parameters. Strawberry and selenium, when administered individually, significantly attenuated memory impairment, oxidative stress, neuroinflammation, and apoptosis, as evidenced by marked normalization of *Nrf2*/*HO-1*, TAC, SOD, MDA, *TLR4*/*NF-κB*/TNF-α, *NLRP3*/*CASP-1*/IL-1β, and *BAX*/*Bcl-2* signaling. Although the concentrations of active constituents were not quantified in brain tissue and direct confirmation of blood–brain barrier penetration is lacking, the magnitude and direction of the observed changes in central oxidative, inflammatory, and apoptotic markers in our in vivo model strongly suggest that bioactive metabolites from these extracts exerted CNS-level actions. In our view, this pattern supports the hypothesis that phenolic- and micronutrient-rich interventions can modulate key pathological pathways in AD beyond simple antioxidant effects.

Notably, the combined strawberry–lemon treatment (S/L) produced the most pronounced and coherent neuroprotective profile, surpassing the effects of each individual treatment across all endpoints assessed. In addition to superior enhancement of cognitive performance and restoration of neurotransmitter balance, S/L treatment more effectively reduced oxidative stress and neuroinflammatory indices and robustly downregulated Alzheimer’s disease–specific biomarkers, including BACE1, Aβ, and p-Tau. The histological findings, showing near-complete preservation of cortical and hippocampal architecture in the S/L group, reinforce the concept that this combination provides integrated structural and functional protection against neurodegeneration. Based on these observations, we propose that the S/L regimen may exert synergistic effects arising from complementary phytochemical compositions and improved bioavailability, and we consider this combination to be a particularly promising candidate for further mechanistic and translational studies. Future studies should specifically address brain pharmacokinetics and blood–brain barrier transport of key metabolites to substantiate these interpretations.

Acidic conditions are pivotal for enhancing the extraction efficiency of phenolic plant metabolites. Flavonoids and tannins are often more effectively extracted using acidified organic solvents, which not only improves their solubility but also aids in stabilizing these compounds against oxidation [56]. Furthermore, mildly acidic environments promote glycoside hydrolysis, releasing bioactive aglycones [57]. Therefore, adjusting the pH of the extraction medium is a key parameter for optimizing the recovery and integrity of the targeted phytochemicals.

Lemon juice can be effectively used as a natural acidic medium for the extraction of plant metabolites, serving as a practical and sustainable alternative to pure citric acid. While citric acid is a single organic acid commonly used to acidify extraction solvents, lemon juice offers a more complex matrix containing citric acid, minor organic acids, and flavonoids. This unique composition may provide synergistic effects that enhance the solubility, stability, and antioxidant protection of sensitive phytochemicals during their extraction. Moreover, the use of lemon juice aligns with green chemistry principles, promoting the use of biodegradable, food-grade, and cost-effective solvents derived from renewable resources [58]. Accordingly, lemon juice serves not only as an acidifying agent but also as a source of endogenous organic acids, ascorbic acid, and citrus-derived phytochemicals that function as natural antioxidant co-solvents and modulate the solvent polarity and pH. These features are expected to enhance both the extraction efficiency and physicochemical stability of phenolic constituents, consistent with previous reports on ascorbic- and citric-acid–mediated improvement of polyphenol recovery and preservation in green tea and fruit-based systems [59,60].

In this study, we demonstrated that using lemon juice as a catalyst effectively facilitated the extraction of phenolic compounds from strawberries. Interestingly, quantitative analysis revealed that the concentrations of gallic acid, ellagic acid, and chlorogenic acid increased by factors of 7.7, 2.1, and 1.2, respectively, when comparing S and S/L samples. This enhancement may be attributed to the acidic conditions, which facilitate the breakdown of ellagic acid into simpler phenolic compounds, such as gallic acid, particularly when oxidation or hydrolysis is encouraged. Hence, acid-solvent extraction holds great promise for the eco-efficient production of EA from plant materials.

According to the phytochemical quantitative analysis of the S/L extract, the most bioactive samples, gallic acid, chlorogenic acid, ellagic acid, rutin, syringic acid, naringenin, catechin, caffeic acid, *p*-coumaric acid, rosmarinic acid, quercetin, and cinnamic acid, were identified as the major compounds (Supplementary File, Figure S1).

The results of this study align with existing evidence that underscores the neurotherapeutic potential of gallic acid (GA) in the prevention and management of AD. GA is a naturally occurring polyphenol with potent antioxidant, anti-inflammatory, and metal-chelating properties, which are critical for mitigating neurodegenerative processes [61]. Previous studies have demonstrated its capacity to modulate key pathological mechanisms, including neuroinflammation, oxidative stress, and impaired synaptic plasticity, which are central to AD progression [62]. In particular, GA has been shown to delay disease onset in AlCl_3_-induced AD models, indicating its potential as a preventive agent [63]. Furthermore, dietary supplementation with GA has led to notable reductions in hippocampal damage and oxidative stress, and improvements in cognitive performance and histopathological outcomes [64]. Its interaction with amyloid-beta (Aβ) peptides, specifically in inhibiting their aggregation, highlights its relevance in targeting amyloidogenic pathways implicated in AD pathogenesis [65]. Additional studies have shown that GA can restore neurotransmission and memory function by attenuating neuronal damage in dementia-like conditions [66]. Moreover, the inhibition of the p38/MAPK signaling pathway by GA suggests a molecular mechanism through which it may exert neuroprotective effects [67]. These pleiotropic actions, including its antioxidant and anti-inflammatory capacities, support the therapeutic value of GA in preserving synaptic function and mitigating Aβ toxicity [68].

Within this framework, the marked difference in GA content between strawberry (S) and strawberry–lemon (S/L) extracts observed in our phytochemical profiling is particularly noteworthy. GA was identified as a major phenolic acid in both extracts but was substantially more abundant in the S/L extract, which coincided with the most pronounced improvements in oxidative stress markers, inflammatory mediators, and AD-related biomarkers in vivo. The superior normalization of *Nrf2*/*HO-1* signaling, attenuation of *TLR4*/*NF-κB*- and *NLRP3*-associated pathways, and more robust reductions in Aβ and p-Tau in the S/L-treated group are therefore plausibly linked, at least in part, to its higher GA content, acting in concert with other co-extracted polyphenols enriched by lemon juice-assisted extraction. From our perspective, these findings suggest that GA may be a key driver of the enhanced neuroprotective profile of the S/L regimen, emphasizing the importance of matrix- and process-dependent modulation of phenolic composition. However, GA was not administered as an isolated compound, and neither its free form nor its metabolites were quantified in the brain tissue. Future studies employing GA-enriched or GA-depleted fractions, alongside targeted brain pharmacokinetic and mechanistic studies, will be essential to disentangle its specific contribution from that of other S/L phytoconstituents and to validate GA as a critical bioactive determinant of the observed therapeutic effects.

Ellagic acid (EA) is a phenolic compound commonly found in plants that is associated with numerous health benefits. It is derived from ellagitannins, which are particularly abundant in berries. The health benefits associated with EA-rich foods are thought to involve various protective mechanisms at the cellular level. The classification of strawberries as functional foods is substantiated by evidence of EA’s biological effects of EA, which include neuroprotective, anti-inflammatory, antidiabetic, cardioprotective, and prebiotic properties [69]. According to Khodadadi and Nasri [70], strawberries are considered functional foods because of their high concentration of EA and its precursors. EA has been extensively investigated for its antioxidative, anti-inflammatory, and anti-amyloidogenic properties, which collectively contribute to its neurotherapeutic effects. Experimental data have demonstrated that EA modulates oxidative stress by enhancing endogenous antioxidant defense systems, such as superoxide dismutase activity, thereby reducing neuronal damage, particularly in vulnerable brain regions, such as the entorhinal cortex [71]. Furthermore, EA has been shown to improve learning and memory performance in preclinical AD models, with effects attributed to its capacity to attenuate neuroinflammation, inhibit amyloid beta (Aβ) aggregation, and preserve synaptic integrity [72,73]. More recent investigations have confirmed that EA significantly reduces Aβ deposition, suppresses pro-inflammatory signaling, and restores redox balance, thereby ameliorating the key pathological features of AD [74,75]. In addition to these mechanistic insights, EA has been proposed as a multitarget therapeutic agent capable of modulating mitochondrial function, microglial activity, and neuronal apoptosis [76,77].

Phenolic acid compounds in the S/L extract could also contribute to diminishing AD. Syringic acid has been shown to modulates cholinesterase activity and may complement other phenolic compounds in reducing cognitive decline [78]. Rosmarinic acid exhibits strong anti-amyloidogenic properties and inhibits acetylcholinesterase activity, both of which are crucial for slowing the progression of Alzheimer’s [79,80]. In addition to the antioxidant and anti-inflammatory properties of cinnamic acid derivatives, they demonstrated inhibitory activity against both human acetylcholinesterase and human butyrylcholinesterase. These enzymes are involved in the breakdown of acetylcholine, a neurotransmitter that is deficient in patients with Alzheimer’s disease. Inhibiting these enzymes can increase acetylcholine levels, potentially improving the cognitive function of affected individuals [81]. Caffeic acid alleviates learning deficits, inhibits acetylcholinesterase (AChE), and modulates neuroinflammation and oxidative stress in AD-like disease (AD)-like pathologies [82]. *p*-Coumaric acid enhances long-term potentiation, restores memory in scopolamine-induced amnesia models, and promotes neurogenesis via BDNF signaling [83,84]. These findings support the therapeutic potential of phenolics, particularly as multitarget agents for AD intervention.

Flavonoid compounds quantified in the S/L extract, such as chlorogenic acid (CGA), contribute to cognitive improvement through acetylcholinesterase inhibition and ROS scavenging, particularly in oxidative stress-induced models [24]. Increasing evidence from both preclinical and clinical studies underscores the potential of CGA as a neurotherapeutic agent in Alzheimer’s disease. Nguyen et al. [85] highlighted the promising neuroprotective agent with potential therapeutic relevance for Alzheimer’s disease. This compound exerts its effects by reducing amyloid-beta aggregation, inhibiting tau hyperphosphorylation, and modulating key signaling pathways, such as PI3K/Akt, MAPK, and *GSK3β*. CGA also demonstrates strong antioxidant and anti-inflammatory activities and can cross the blood-brain barrier, supporting its role in preserving cognitive function. Clinical research further validates these outcomes, with randomized controlled trials indicating improved cognitive performance in individuals with mild cognitive impairment after CGA supplementation [86]. Recent innovations have integrated CGA into nanocarrier-based delivery systems to address bioavailability issues, thereby enhancing its therapeutic potential for targeting neurodegenerative processes [87].

In addition, rutin glycosides and catechins inhibit oxidative stress and protect against β-amyloid-induced neurotoxicity by enhancing endogenous antioxidant systems [88,89]. Moreover, naringenin crosses the blood-brain barrier and modulates neuroinflammatory pathways, enhancing neuronal survival and cognitive performance [90].

In establishing a correlation between biological and chemical findings, the pharmacological effect of S/L on neurotransmitters indicated an increase in dopamine, with a 3.4-fold increase compared to the control. This increase may be attributed to the presence of L-beta-homophenylalanine in strawberry/lemon extracts. L-beta-homophenylalanine is structurally analogous to phenylalanine and influences dopamine metabolism, neurotransmitter balance, and protein synthesis, suggesting its role in the metabolic dysregulation of Alzheimer’s [91,92]. This finding is consistent with a previous study indicating that antioxidant diets containing strawberry extract can prevent aging-related neurochemical changes in rats by enhancing dopaminergic function and dopamine release, thereby improving motor behavior and learning [93,94,95].

**Figure 12:**
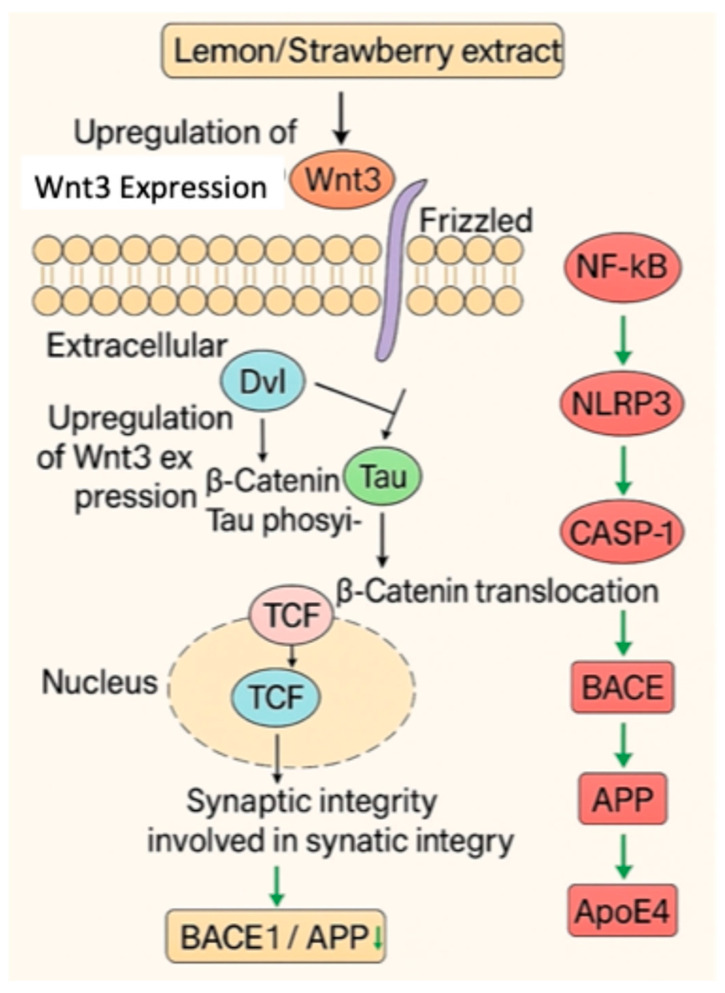
Mechanistic pathways underlying the neuroprotective effects of S/L extract in an Alzheimer’s disease model.

Chromatographic fingerprinting and comprehensive phytochemical profiling of lemon juice-assisted strawberry extract (S/L) indicated that the identified metabolites interact with key pathological mechanisms of Alzheimer’s disease, including oxidative stress, amyloid-beta aggregation, and cholinergic dysfunction (Figure 12). Notably, lemon juice functions not only as an extraction medium but also as a bioactive modulator, promoting chemical transformations that enhance the structural diversity and bioactivity of phytochemicals. This co-extraction approach significantly enhances the neuroprotective and pharmacological potentials of the resulting S/L extract.

Beyond the antioxidant effects of the S/L extract, the findings indicated that it (i) suppressed key inflammatory markers (TNF-α, IL-1β, *NF-κB*, *NLRP3*), supporting prior evidence of polyphenols acting as *NLRP3* inflammasome inhibitors. (ii) Downregulated amyloidogenic proteins (BACE1, APP, *p*-Tau) and increased LRP1, a receptor critical for Aβ clearance, indicating strong modulation of Aβ metabolism. (iii) Restored Wnt3/β-catenin signaling with concurrent *GSK3β* suppression, highlighting the reversal of tau hyperphosphorylation and synaptic loss. This convergence on multiple AD-related pathways distinguishes the S/L extract as a multitarget therapeutic candidate. The co-extracted compounds likely engage in synergistic interactions, thereby enhancing their therapeutic potential. In particular, the upregulation of the Wnt3/β-catenin pathway and the concomitant inhibition of *GSK3β* suggest a role in promoting synaptic plasticity and attenuating hyperphosphorylation of tau. Additionally, the modulation of the Wnt3/β-catenin/*GSK3β*, *NF-κB*/*NLRP3*, and BACE1/APP signaling pathways highlights the mechanistic significance of phytochemical synergy (Figure 12). These findings underscore the therapeutic relevance of the S/L extract as a multitargeted complementary intervention against aluminum-induced neurotoxicity and Alzheimer-like neurodegeneration.

In summary, the neuroprotective effects of strawberry and lemon extracts support their potential as candidates for developing natural therapeutic strategies for neurodegenerative disorders. To advance these findings toward clinical application, it is necessary to define doses that are relevant to humans in the future. Using the Reagan–Shaw interspecies dose-conversion approach [96], the rat dose employed in this study (200 mg/kg) corresponds to an estimated human-equivalent dose of 32.4 mg/kg. An important limitation of the present study is that the ability of the extracted metabolites to cross the blood–brain barrier was not directly assessed. Compared with previous in vivo studies that have predominantly examined isolated phenolic compounds or single-fruit extracts in Alzheimer’s models, the present work indicates that the strawberry–lemon co-extract achieves a broader and more coordinated modulation of oxidative, inflammatory, apoptotic, and amyloidogenic pathways, accompanied by near-complete preservation of hippocampal and cortical architecture. Furthermore, a key novel aspect of this study is the implementation of a lemon juice-assisted green extraction strategy, which, in combination with metabolomic characterization, yields a phytochemically enriched co-extract with superior neuroprotective efficacy relative to the individual treatments.

The observed behavioral and molecular improvements are compatible with the notion that at least a subset of low-molecular-weight phenolic acids and flavonoids can reach the brain, as previously reported for several dietary phenolics and their metabolites in experimental models and human-relevant systems [97]. Nonetheless, this remains indirect evidence and does not replace dedicated permeability and brain distribution studies. Therefore, future investigations should incorporate blood–brain barrier transport and brain pharmacokinetic profiling, alongside the evaluation of the human-equivalent dose in rigorously designed preclinical and clinical studies, to more robustly substantiate the translational potential of these extracts in neurodegenerative disease management.

## 4. Material and Methods

### 4.1. Material

Fresh strawberries (*Fragaria ananassa*) and lemons (*Citrus limon*) were obtained from a local market in Cairo, Egypt. Selenium and aluminum chloride (AlCl_3_·6H_2_O, CAS No: 7784-13-6) were obtained from Sigma-Aldrich (St. Louis, MO, USA). All chemicals and reagents used were of analytical grade.

### 4.2. Extracts Preparation

Three types of extracts were prepared: strawberry (S), lemon (L), and lemon-assisted strawberry (S/L). All procedures were performed under controlled laboratory conditions to ensure reproducibility.

#### 4.2.1. Strawberry Extract (S)

Fresh strawberries (2.0 kg) were obtained from a local market, thoroughly washed and cut into small pieces. The chopped fruits, together with their endogenous juice, were diluted with 2 L distilled water and acidified with 8.3 mL glacial acetic acid to adjust the pH to 2.65. This maceration procedure was repeated twice under the same conditions with intermittent manual stirring (total maceration time 72 h) until exhaustive extraction. The macerates were filtered and concentrated under reduced pressure to obtain 500 mL of crude strawberry extract. The crude extract was mixed with 2.0 L ethanol, under 4 °C and the filtrate reconcentrated for further purification. The final filtrate was concentrated in a lyophilizer to yield the dry S extract (99.3 g), corresponding to an extraction yield 4.97 g dry extract per 100 g fresh strawberries.

#### 4.2.2. Lemon Extract (L)

Fresh lemons (250 g) were manually pressed, and the juice was filtered to remove seeds and pulp. The clarified juice was diluted with distilled water to a final volume of 1.0 L, thoroughly mixed, and concentrated under reduced pressure at 40 °C using a rotary evaporator until a viscous residue was obtained. The residue was further dried under reduced pressure to constant weight to afford the L extract. The final dry mass of the L extract was 15.0 g, corresponding to an extraction yield of 6.0 g dry extract per 100 g fresh lemons.

#### 4.2.3. Lemon-Assisted Strawberry Extract (S/L)

For the S/L extract, fresh strawberries (2 kg; washed, hulled, and cut into small pieces as described above) with 1.0 L distilled water and 1.0 L freshly squeezed lemon juice (250 g fresh lemons; pH ≈ 2.7), corresponding to a strawberry: lemon-juice ratio of 2 kg: 2.0 L (*w*/*v*). The mixture was macerated at 25 °C for 24 h; this maceration step was then repeated for 7 days till exhausted extraction. The total macerates were concentrated under reduced pressure to obtain 500 mL of crude S/L extract. The crude extract was then mixed with 2.0 L of ethanol at 4 °C, and the filtrate was reconcentrated under reduced pressure for further purification to yield the S/L extract (210 g), corresponding to an extraction yield of 10.5 g of dry extract per 100 g of fresh strawberries.

### 4.3. Phytochemical Characterization of S, L and S/L Extracts

#### 4.3.1. Nutrient Composition of Extracts

##### Total Carbohydrate Determination

The total carbohydrate content was determined according to the method described by Sadasivam et al. [98]. A dried sample (0.2 g) was hydrolyzed with 10 mL of 0.1 N H_2_SO_4_ in a sealed tube and incubated at 105 °C overnight. The resulting hydrolysate was filtered and diluted to a final volume of 100 mL. Glucose standards ranging from 20 to 640 ppm were prepared for calibration. For the colorimetric assay, 100 μL of each standard or sample was mixed with 1 mL of 5% phenol and 5 mL of concentrated H_2_SO_4_. After 15 min of incubation, the absorbance was measured at 490 nm. The carbohydrate content was calculated from the standard curve and expressed as mg/g dry weight.

##### Lipid Content Determination

Lipid content was quantified using a modified Soxhlet extraction method [99]. A precisely 5.0 g oven-dried sample was weighed and transferred into a mortar. The sample was thoroughly homogenized with 50 mL of petroleum ether for 10 min to facilitate lipid solubilization. The homogenate was filtered, and the filtrate was collected in a pre-cleaned, pre-weighed dry round-bottom flask (W_1_). The solvent was evaporated in a water bath to dryness, and the flask was cooled in a desiccator to ambient temperature and re-weighed (W_2_). The lipid content was determined gravimetrically using the following equation:

$$\mathrm{Lipid}\;(\%)\;=\;(W2\;-\;W1)\;\times \;\frac{100}{5}$$

##### Determination of Protein Content

Protein content was quantified using the Biuret colorimetric assay as described by Keppy et al. [100]. The Biuret reagent was prepared by dissolving copper (II) sulfate (5 g), sodium potassium tartrate (9 g), and potassium iodide (5 g) in 0.2 N sodium hydroxide solution and adjusting the final volume to 1 L. A 3000 ppm bovine serum albumin (BSA) stock solution was prepared and serially diluted to generate standards ranging from 200 to 3000 ppm. For analysis, 1.0 g of dried sample was homogenized in 2.0 mL of 0.85% saline solution, followed by the addition of 3.0 mL of Biuret reagent. After incubation at room temperature for 30 min, the absorbance was measured at 540 nm. A blank was prepared by replacing the sample with saline solution. Protein concentration was determined using a standard curve and expressed as a percentage of dry weight.

##### Total Soluble Sugar Determination

Total soluble sugars were determined using the phenol-sulfuric acid method described by Dubois et al. [101]. A dried sample (0.2 g) was extracted by grinding in boiling distilled water, filtered, and diluted to 100 mL. Glucose standards (20–640 ppm) were prepared from 1000 ppm and 100 ppm stock solutions. For the assay, 100 μL of blank, standard, or sample was mixed with 1 mL of 5% phenol and 5 mL of concentrated H_2_SO_4_. After incubation for 15 min, the absorbance was measured at 490 nm. The soluble sugar content was calculated from the standard curve and expressed as mg/g dry weight.

##### Reducing and Non-Reducing Sugar Determination

The reducing sugar content was determined using the DNS method described by Miller [102]. A 0.5 g sample was extracted with 80% ethanol and centrifuged. Aliquots of the extract or glucose standards (20–640 ppm) were reacted with DNS reagent, heated in a boiling water bath, and treated with sodium potassium tartrate. The absorbance was measured at 515 nm. The reducing sugar content (mg/g) was calculated from a glucose standard curve. The non-reducing sugar content was calculated by subtracting the reducing sugars from the total soluble sugar content [101].

#### 4.3.2. Quantitative Determination of Total Phenolic Content

The total phenolic content was determined using the Folin–Ciocalteu method [103,104]. One milliliter of the extract was dissolved in 2 mL of methanol. An aliquot (500 μL) of the extract was mixed with 2.5 mL of Folin–Ciocalteu reagent (diluted ten-fold) and 2.5 mL of sodium carbonate solution (75 g/L). The mixture was vortexed for 10 s and left to stand at 25 °C for 2 h. Absorbance was measured at 765 nm against a reagent blank. The results were expressed as milligrams of gallic acid equivalent (GAE) per gram of extract.

#### 4.3.3. HPLC Analysis

High-performance liquid chromatography (HPLC) analysis was performed using an Agilent 1260 Infinity series chromatograph (Agilent Technologies, Santa Clara, CA, USA) equipped with a Zorbax Eclipse Plus C8 column (4.6 mm × 250 mm, 5 µm). The mobile phase comprised water (A) and 0.05% trifluoroacetic acid in acetonitrile (B) at a flow rate of 0.9 mL/min. A linear gradient was employed as follows: 0–1 min (82% A), 1–11 min (75% A), 11–18 min (60% A), 18–22 min (82% A), and 22–24 min (82% A). The multiwavelength detector was set to 280 nm. The injection volume was 5 µL, and the column temperature was maintained at 40 °C.

#### 4.3.4. Qualitative Analysis and Fingerprinting Using LC-ESI-MS/MS Analysis

Sample analysis was performed using liquid chromatography–electrospray ionization–tandem mass spectrometry (LC-ESI-MS/MS) with an ExionLC™ AC system (AB SCIEX, Framingham, MA, USA) for separation and a SCIEX Triple Quad 5500+ MS/MS system (Singapore) equipped with electrospray ionization (ESI) for detection. Separation was accomplished using a Poroshell 120 EC-C18 column (Agilent, Wilmington, DE, USA) (3.0 × 100 mm, 2.7 µm). The mobile phases consisted of two eluents: A, 0.1% formic acid in water, and B, acetonitrile (LC-grade). The injection volume was set to 5 µL. Negative ionization mode was employed with the following mass spectrometer parameters: curtain gas at 25 psi, Ion Spray voltage at −4500, source temperature at 400 °C, and ion source gases 1 and 2 at 55 psi.

### 4.4. Anti-Alzheimer In Vivo Model

Sixty male Sprague-Dawley rats (300–320 g) were obtained from Nile Co. (Cairo, Egypt) and acclimatized for one week under standard laboratory conditions (25 ± 1 °C, 50 ± 5% relative humidity, and a 12-h light/dark cycle) at the Faculty of Pharmacy, Al-Azhar University. The animals were housed in stainless steel cages with free access to standard rodent chow and water ad libitum. All experimental procedures were performed in accordance with international ethical standards and were approved by the Institutional Animal Care and Use Committee (IACUC) of the Faculty of Pharmacy, Al-Azhar University (Approval No. 355/2022). This study complied with the Animal Research: Reporting of In Vivo Experiments (ARRIVE) guidelines and adhered to the principles of the NIH Guide for the Care and Use of Laboratory Animals (NIH Publication No. 8023, revised 1978).

Animals were randomly allocated to six experimental groups (*n* = 10 per group) and treated for five consecutive weeks as follows:

- Group 1 (Normal Control): Received normal saline (1 mL/kg, i.p.).
- Group 2 (AD Model): Administered aluminum chloride (AlCl_3_; 70 mg/kg, i.p.) daily for 5 weeks to induce AD-like conditions [105].
- Group 3: Received AlCl_3_ (70 mg/kg, i.p.) along with strawberry extract (200 mg/kg, p.o.) daily for 5 weeks [106].
- Group 4: Received AlCl_3_ and lemon juice (200 mg/kg, p.o.) daily for 5 weeks [107].
- Group 5: Co-administered AlCl_3_ with both strawberry and lemon extracts (1:1 ratio) (200 mg/kg each, p.o.) daily for 5 weeks.
- Group 6 (Positive Control): Received AlCl_3_ and selenium (1 mg/kg, p.o.) [108] daily for 5 weeks.

On day 32, the Morris water maze (MWM) test was started until day 35, with four days of training trials. Twenty-four hours after the final treatment (day 36), a probe test of the MWM test was performed, followed by the Y-maze test on day 37, and finally, the conditioned avoidance test was performed on day 38. Twenty-four hours after the final treatment. The animals were anesthetized with ketamine (80 mg/kg, i.p.) and euthanized via cervical dislocation. Brain tissues were carefully excised, rinsed with phosphate-buffered saline (PBS; pH 7.4), and immediately prepared for histological, biochemical, and molecular investigations.

### 4.5. Evaluation of Behavioral Parameters

#### 4.5.1. Y-Maze Spontaneous Alternation (SAP) Test

Spatial working memory, a fundamental aspect of short-term memory, was assessed using the spontaneous alternation performance (SAP) paradigm. The test was conducted in a black wooden Y-maze apparatus comprising three identical arms (designated A, B, and C) radiating at 120° from a central equilateral triangular zone. Each rat was placed at the entrance of one arm and allowed to explore the maze freely for 8 min. An arm entry was defined as the placement of all four limbs, including the hind paws, within an arm of the apparatus. The percentage of spontaneous alternation, which is indicative of spatial working memory integrity, was calculated using the following equation:

SAP (%) = [Number of alternations/(Total number of arm entries − 2)] × 100

This metric quantifies the tendency of an animal to enter all three arms sequentially, reflecting an intact spatial working memory function [109].

#### 4.5.2. Morris Water Maze Test (MWM)

Spatial learning and memory capabilities were evaluated using the Morris Water Maze (MWM) test, as previously described [110]. A circular water tank (150 cm in diameter and 60 cm in height) was filled to a depth of 30 cm with tap water maintained at 25 ± 2 °C. Non-toxic white paint was added to obscure visual cues and render the water opaque. The pool was conceptually divided into four quadrants (north, south, east, and west), with a submerged escape platform (10 cm in diameter) placed 2 cm below the water surface in a fixed location at the center of one of the quadrants. The position of the platform was constant throughout the training sessions. Overhead video tracking was used to monitor and record the swimming trajectories of the animals. During each trial, the rats were released into the pool from a randomly assigned starting point in one of the quadrants, facing the pool wall, and allowed to navigate toward the hidden platform. Each animal underwent four training trials per day for three days. The maximum trial duration was 60 s; if the rat failed to locate the platform within this period, it was gently guided to it and allowed to rest for 20 s before the next trial. The primary outcome measure was escape latency, which was defined as the time required to locate the hidden platform. On the fourth day, a probe trial was conducted by removing the platform and allowing the animals to swim freely for 60 s. The time spent in each quadrant was recorded to assess spatial memory retention.

#### 4.5.3. Conditioned Avoidance Test (CA)

The conditioned avoidance (CA) test was used to assess associative learning and memory consolidation under stress-inducing conditions [111]. A custom-designed wooden apparatus consisting of five interconnected chambers was used in this study. Four of the chambers had electrified floors connected to a pulse generator (50 volts, 25 pulses per second), while the fifth chamber, constructed with a glass floor, served as the safe zone. Transparent movable glass partitions allowed for controlled transition between the chambers. The rats were pre-trained one day prior to testing. Training consisted of a 5-s auditory signal (conditioned stimulus), followed immediately by a 5-s mild foot shock (unconditioned stimulus) in the electrified chambers. Post-treatment testing was conducted over two consecutive days using the same paradigms. The number of trials required for each rat to reach the safe chamber within 5 s of the conditioned auditory stimulus, thereby avoiding the shock, was recorded. This measure served as an index of learning efficiency and short-term memory retention.

### 4.6. Preparation of Tissue Samples

Following euthanasia, the brain tissues were carefully excised and rinsed with ice-cold saline to remove residual blood. For histopathological evaluation, four brains from each experimental group were fixed in 10% neutral-buffered formalin and embedded in paraffin. The remaining six brains were bisected and processed for biochemical and molecular analysis. One portion was homogenized in ice-cold 50 mM Tris-HCl buffer (10% *w*/*v*) containing 300 mM sucrose (pH 7.4), followed by centrifugation at 1800× *g* for 10 min at 4 °C. The resulting supernatant was collected and reserved for subsequent assays. The second portion was snap-frozen and stored at −80 °C for later use in quantitative real-time polymerase chain reaction (qRT-PCR) analysis [18].

### 4.7. Biochemical Analyses

#### 4.7.1. Colorimetric Analysis

Total antioxidant capacity (TAC), superoxide dismutase (SOD) activity, and malondialdehyde (MDA) levels were quantified in brain tissue homogenates using colorimetric assay kits (Bio-Diagnostic, Cairo, Egypt). Absorbance was measured at 510 nm for TAC, 560 nm for SOD, and 534 nm for MDA.

#### 4.7.2. Fluorometric Assays

Brain monoamine levels, including dopamine (DA), norepinephrine (NE), and serotonin (5-HT), were quantified using fluorometric assays with commercially available kits (Sigma-Aldrich, St. Louis, MO, USA) according to previously established protocols [18]. The detection method involves the oxidation of monoamines to their respective adrenochromes, which are then converted to fluorescent adrenolutins. Fluorescence was measured at specific excitation/emission wavelengths of 320/480 nm for DA, 380/480 nm for NE, and 355/470 nm for 5-HT.

#### 4.7.3. Enzyme-Linked Immunosorbent Assay (ELISA)

The concentrations of tumor necrosis factor-α (TNF-α) and interleukin-1β (IL-1β) in brain tissues were quantified using enzyme-linked immunosorbent assay (ELISA) kits: Quantikine^®^ Rat TNF-α ELISA Kit (Catalog No. RTA00; R&D Systems, Minneapolis, MN, USA) and IL-1β ELISA Kit (Catalog No. CSB-E08055r (CusaBio Life Science Inc., Wuhan, China). ELISA kits were purchased from MyBioSource, Inc. (San Diego, CA, USA) were employed to measure the levels of various neuroinflammatory and neurodegenerative markers, including amyloid precursor protein (APP), amyloid-β (Aβ), brain-derived neurotrophic factor (BDNF), acetylcholinesterase (AChE), β-catenin, chitinase-3-like protein 1 (*CHI3L1*), phosphorylated tau (*p*-Tau), Wnt3, apolipoprotein E4 (ApoE4), β-site amyloid precursor protein cleaving enzyme 1 (BACE1), and low-density lipoprotein receptor-related protein 1 (LRP1). All assays were performed according to the manufacturer’s protocols.

#### 4.7.4. Real-Time Quantitative Polymerase Chain Reaction (RT-qPCR)

Real-time quantitative polymerase chain reaction (RT-qPCR) was conducted to assess the mRNA expression levels of key apoptotic, inflammatory, and antioxidant genes, including *Bcl-2*–associated X protein (*BAX*), B-cell lymphoma 2 (*Bcl-2*), caspase-1 (*CASP-1*), glycogen synthase kinase-3β (*GSK3β*), heme oxygenase-1 (*HO-1*), nuclear factor erythroid 2–related factor 2 (*Nrf2*), nuclear factor kappa B (*NF-κB*), nucleotide-binding domain leucine-rich repeat, toll-like receptor 4 (*TLR4*), and pyrin domain–containing protein 3 (*NLRP3*). The housekeeping gene, *β-actin*, was used as an internal control. Total RNA was extracted from brain tissues using the Qiagen RNeasy tissue extraction kit (Qiagen, Germantown, MD, USA), and cDNA was synthesized using the Sense Rapid cDNA Synthesis Kit (Catalog No. BIO-65053), according to the manufacturer’s protocol and the method described by Livak et al. [112]. RT-qPCR was performed using an Applied Biosystems StepOnePlus™ Real-Time PCR System. Relative gene expression was calculated using the 2^−ΔΔCT^ method. The primer sequences used for amplification are listed in Table 4.

### 4.8. Histopathological Evaluation

Rat brain tissues were carefully excised, fixed in 10% neutral-buffered formalin, and embedded in paraffin. Serial sections were cut at a thickness of 4 μm and stained with hematoxylin and eosin (H&E) for histopathological evaluation. The stained sections were examined under a light microscope, and representative photomicrographs were captured at 40× magnification [113].

Neuronal integrity was assessed using a semi-quantitative scoring system based on the number of non-intact (degenerating) neurons per field, identified by cytoplasmic eosinophilia, nuclear pyknosis, and cell shrinkage. In each brain region (corpus callosum, striatum, frontal cortex, and subiculum), five non-overlapping fields per animal were examined at ×400 magnification. Damage severity was categorized as follows: score 0 (0–5 non-intact neurons), score 1 (6–20), score 2 (21–50), and score 3 (>50), reflecting no, mild, moderate, and severe degeneration, respectively, respectively. The regional scores were summed to yield a composite neurodegeneration score (range: 0–12) per animal, representing cumulative damage across all regions. Data are expressed as mean ± standard deviation (SD) and statistically analyzed using one-way ANOVA followed by Tukey’s post hoc test, with *p* < 0.05 considered significant (GraphPad Prism) [114].

### 4.9. Statistical Analysis

All data are presented as mean ± standard error of the mean (SEM). Statistical analyses were performed using one-way analysis of variance (ANOVA), followed by Tukey’s post hoc test to determine the intergroup differences. Analyses were conducted using GraphPad Prism (version 8.0; GraphPad Software, San Diego, CA, USA). Statistical significance was set at *p* < 0.05. In addition, effect sizes (η^2^) were calculated to evaluate the magnitude of the differences among the treatment groups.

## 5. Conclusions

This study demonstrates that strawberry extract, enhanced through lemon juice-assisted extraction (S/L), confers significant neuroprotective effects in an AlCl_3_-induced rat model of Alzheimer’s disease. The co-extracted S/L treatment markedly improved cognitive performance, reduced oxidative and inflammatory markers, and modulated key apoptotic and amyloidogenic pathways more effectively than either extract administered alone. Phytochemical analysis revealed elevated levels of bioactive polyphenols, particularly gallic acid, ellagic acid, and chlorogenic acid, in the S/L extract, underscoring the efficacy of lemon juice as a natural acidifier that enhances the extraction and bioactivity of neuroprotective phenolics. Mechanistically, the S/L extract activated the *Nrf2*/*HO-1* antioxidant pathway and attenuated neuroinflammation by downregulating the *TLR4*/*NF-κB* axis, along with significant reductions in TNF-α, IL-1β, and the neuroinflammatory tissue injury marker *CHI3L1*. The treatment also inhibited the BACE1/APP/Aβ and *p*-Tau cascades, restored apoptotic balance by regulating the *BAX*/*Bcl-2* axis, and suppressed inflammasome activation by downregulating *NLRP3* and caspase-1. Additionally, it reactivated the Wnt3/β-catenin signaling pathway and inhibited *GSK3β*, thereby supporting improved neurogenesis and tau regulation. These multitargeted effects translate into enhanced neurotransmitter balance, improved behavioral performance, and preserved neuronal architecture. These findings highlight the therapeutic potential of lemon juice-assisted strawberry extraction as a functional food-based strategy for the complementary management of Alzheimer’s disease. Although the outcomes are promising, this study is limited by its clinical scope and lack of pharmacokinetic evaluation. Future research should explore the bioavailability and translational potential of the extract in clinical settings. Nonetheless, this study underscores the value of polyphenol-rich dietary interventions and green extraction techniques in advancing neuroprotective strategies against Alzheimer’s disease.

## Figures and Tables

**Table 1:** Secondary metabolites identified from Strawberry Fruit (S) extract using LC-MS/MS in ESI-Negative ionization mode.

ID	Metabolites	Chemical Formula	RT	M.wt	*m*/*z*	Mass Fragments
1	Pentonic acid	C_5_H_10_O_6_	1.61	166.04	165.03	103.99, 120.96, 136.99, 149.08, 165.03
2	Gallic acid	C_7_H_6_O_5_	1.90	170.02	169.09	124.99, 141.99
3	2-Deoxyerythropentono-1,4-lactone	C_5_H_8_O_4_	2.26	132.04	131.02	102.92, 113.01, 118.96, 129.99, 131.03
4	*p*-Coumaric acid	C_9_H_8_O_3_	4.72	164.04	163.00	119.04, 146.91
5	Cyanidin-3-*O*-hexoside	C_21_H_21_O_11_^+^	5.26	449.10	448.94	125.05, 179.05, 259.09, 286.97
6	Isozonarol	C_21_H_30_O_2_	5.57	314.22	313.04	151.14, 176.95, 194.89, 268.89, 294.62, 312.86
8	Chlorogenic acid	C_16_H_18_O_9_	6.11	354.09	354.99	134.12, 160.09, 175.07, 193.06
9	Roseoside	C_19_H_30_O_8_	6.99	386.19	385.04	153.10, 205.15
10	Pelargonidin-3-glucoside	C_21_H_21_O_10_^+^	7.04	433.11	415.01	100.91, 161.05, 270.07
11	Pelargonidin-3-malonylglucoside	C_24_H_23_O_13_^+^	9.19	519.42	518.04	136.08, 151.14, 375.07
12	Hesperidin	C_28_H_34_O_15_	9.43	610.19	608.96	301.08, 325.06
13	Isoquercitrin	C_21_H_20_O_12_	9.52	464.09	464.04	109.08, 127.10, 208.19, 223.13, 275.14, 292.19, 301.03, 336.18
14	alpha-Bisabolol	C_15_H_26_O	10.77	222.19	221.10	164.18, 205.21, 220.26, 221.16
15	Ellagic acid	C_14_H_6_O_8_	19.71	302.19	301.08	143.19, 217.16, 285.11
16	Peonidin-3-glucoside	C_22_H_23_O_11_	22.28	463.11	461.28	329.08, 461.28
17	Quercetin 3-xyloside-7-glucoside	C_26_H_28_O_16_	26.81	596.14	595.34	435.38, 467.17, 557.26, 595.29

RT, retention time; M.wt, molecular weight; *m*/*z*, precursor ion detected in ESI-negative mode; mass fragments, major diagnostic product ions observed in the MS/MS spectra. The highlighted peak in the corresponding LC–ESI–MS/MS chromatogram represents the base peak (100% relative intensity), and the intensities of the remaining ions are expressed relative to the base peak.

**Table 2:** Secondary metabolites identified from Lemon juice (L) extract using LC-MS/MS using ESI-negative ionization mode.

ID	Metabolites	Chemical Formula	RT	M.wt	*m*/*z*	Mass Fragments
1	Gallic acid	C_7_H_6_O_5_	0.14	170.02	169.01	125.03, 132.96, 150.92
2	Citric acid	C_6_H_8_O_7_	2.41	192.02	174.94	110.95, 118.96, 138.97, 146.99, 159.02, 175.07
3	Vanillic acid	C_8_H_8_O_4_	2.57	168.04	166.99	108.01, 152.03
4	*p*-Coumaric acid	C_9_H_8_O_3_	3.35	164.04	163.01	119.04
5	Citral	C_10_H_16_O	3.81	152.12	151.04	108.01, 120.97, 135.08
6	Linalool	C_10_H_18_O	4.60	154.13	152.98	106.97, 123.01
7	Eriocitrin	C_27_H_32_O_15_	8.16	596.17	594.94	107.03, 151.06, 286.97, 458.99, 594.93
8	Narirutin	C_27_H_32_O_14_	8.90	580.17	578.95	151.04, 271.02, 313.01
9	Hesperidin	C_28_H_34_O_15_	9.33	610.18	609.11	286.05, 301.07, 325.04
10	Apigenin	C_15_H_10_O_5_	10.22	270.05	269.06	123.09, 153.14, 207.15, 251.14
11	Chlorogenic acid	C_16_H_18_O_9_	10.54	354.09	336.99	120.03, 148.06, 177.02, 234.01, 336.99
12	Diosmetin	C_16_H_12_O_6_	10.82	300.05	299.05	282.96, 149.08, 176.08, 277.04, 255.00, 282.96
13	Kaempferol	C_15_H_10_O_6_	13.20	286.04	285.04	133.10, 193.00, 242.08, 270.00
14	Limonin	C_26_H_30_O_8_	13.85	470.19	469.03	229.18, 278.12, 321.22, 381.13
15	Ellagic acid	C_14_H_6_O_8_	19.63	302.19	301.07	143.18, 217.11, 285.12

RT, retention time; M.wt, molecular weight; *m*/*z*, precursor ion detected in ESI-negative mode; mass fragments, major diagnostic product ions observed in the MS/MS spectra. The highlighted peak in the corresponding LC–ESI–MS/MS chromatogram represents the base peak (100% relative intensity), and the intensities of the remaining ions are expressed relative to the base peak.

**Table 3:** Secondary metabolites identified from S/L extract using LC-MS/MS using ESI-negative ionization mode.

ID	Metabolites	Chemical Formula	RT (min)	M.wt	*m*/*z*	Mass Fragments
1	Turanose	C_12_H_22_O_11_	1.06	342.11	340.99	100.86, 113.02, 143.07, 161.06
2	Daphnetin	C_9_H_6_O_4_	1.10	178.02	177.00	110.96, 129.02, 148.95, 158.97
3	4-Methoxy-2,5-dimethyl-3(2H) furanone	C_7_H_10_O_3_	1.40	142.06	140.98	110.99, 122.98, 136.85, 139.03, 140.97
4	D-3-Phenyllactic acid	C_9_H_10_O_3_	1.74	166.06	165.02	100.92, 118.98, 145.00
5	O-trans-Cinnamoyl-b-D-glucopyranose	C_15_H_18_O_7_	2.00	310.10	308.96	123.02, 135.09, 141.08, 151.06, 177.11, 245.96
6	Threitol	C_4_H_10_O_4_	4.78	122.05	121.05	107.98, 118.90, 121.02, 133.94
7	Methyl butyrate	C_5_H_10_O_2_	4.87	102.06	101.06	100.79
8	2-Methylbutanoic acid	C_5_H_10_O_2_	5.75	102.06	101.06	100.78
9	Gallic acid	C_7_H_6_O_5_	5.83	170.02	168.89	125.03, 132.96, 150.94
10	Tormentic acid	C_30_H_48_O_5_	7.40	488.34	487.11	147.05, 183.06, 249.02, 374.54, 424.63, 451.24, 487.10
11	β-Ocimene	C_10_H_16_	8.32	136.12	135.97	105.95, 117.95, 135.97
12	Ellagic acid	C_14_H_6_O_8_	11.88	302.19	301.09	202.08, 229.07, 244.05, 301.09
13	2-Oxobutyric acid	C_4_H_6_O_3_	7.85	102.03	100.95	100.81
14	7,7-Dimethyl-3,4-octadiene	C_10_H_18_	7.93	138.14	137.13	137.98, 155.99
15	Undecane	C_11_H_24_	8.13	156.18	155.18	136.88, 154.93
16	Thymidine	C_10_H_14_N_2_O_5_	9.06	242.08	241.07	110.98, 154.97, 194.99, 222.89, 240.94
17	Cyanidin	C_15_H_11_O_6_^+^	13.34	287.05	286.15	118.09, 238.29, 240.33, 242.28, 286.18, 268.26
18	Chlorogenic acid	C_16_H_18_O_9_	18.06	354.09	353.11	163.19, 177.12
19	Chrysin	C_15_H_10_O_4_	20.94	254.05	253.04	138.06, 152.08, 166.08, 235.06, 253.19
20	2,4-Bis(1,1-dimethylethyl)-phenol	C_14_H_22_O	22.98	206.16	205.11	189.26, 205.17
21	Peonidin-3-glucoside	C_22_H_23_O_11_	23.00	463.11	461.25	279.22, 461.24
22	Pelargonidin-3-malonylglucoside	C_24_H_23_O_13_^+^	24.43	519.10	498.07	452.31, 471.27, 498.07

RT, retention time; M.wt, molecular weight; *m*/*z*, precursor ion detected in ESI-negative mode; Mass fragments, major diagnostic product ions observed in the MS/MS spectra. The highlighted peak in the corresponding LC–ESI–MS/MS chromatogram represents the base peak (100% relative intensity), and the intensities of the remaining ions are expressed relative to this base peak.

**Table 4:** List of primer sequence sets used for RT-qPCR analysis of rat tissues.

Gene	Forward and Backward Sequences	Accession Number
*BAX*	F: 5′-CGGCGAATTGGAGATGAACTGG-3′ R: 5′-CTAGCAAAGTAGAAGAGGGCAACC-3′	NM_031530
*Bcl-2*	F: 5′-CTAGCAAAGTAGAAGAGGGCAACC-3′ R: 5′-TGTGGATGACTGACTACCTGAACC-3′	NM_199267
*Nrf2*	F: 5′-CTCTCTGGAGACGGCCATGACT-3′ R: 5′-CTGGGCTGGGGACAGTGGTAGT-3′	NM_031789
*HO-1*	F: 5′-CACCAGCCACACAGCACTAC-3′ R: 5′-CACCCACCCCTCAAAAGACA-3′	NM_012580
*TLR4*	F: 5′-TCAGCTTTGGTCAGTTGGCT-3′ R: 5′- GTCCTTGACCCACTGCAAGA-3′	NM_019178
*NF-κB*	F: 5′-TTCCTCAGCCATGGTACCTC-3′ R: 5′-CCCCAAGTCTTCATCAGCAT-3′	NM-009045
*CASP-1*	F: 5′-GAACAAAGAAGGTGGCGCAT-3′ R: 5′-GAGGTCAACATCAGCTCCGA-3′	NM_012762
*NLRP3*	F:5′-TGCATGCCGTATCTGGTTGT-3′ R:5′-ACCTCTTGCGAGGGTCTTTG-3′	NM_001191642
*GSK3β*	F: 5′-AGCCTATATCCATTCCTTGG-3′ R: 5′-CCTCGGACCAGCTGCTTT-3′	NM_032080
*CHI3L1*	F: 5′-GAGCTGCTTCCCAGATGCCC-3′ R: 5′-CATGCCATACAGGGTTACGTC-3′	NM_001309820
*β-actin*	F: 5′-CCGTAAAGACCTCTATGCCA-3′ R: 5′-AAGAAAGGGTGTAAAACGCA-3′	NM_031144

## Data Availability

The original contributions presented in this study are included in the article/Supplementary Materials. Further inquiries can be directed to the corresponding authors.

## Supplementary Materials

The following supporting information can be downloaded at: https://www.mdpi.com/article/10.3390/ph18121892/s1, Table S1. HPLC-Based analysis of phenolic compounds identified as area percentage (% Area). (Values represent the relative area percentage of identified phenolic compounds in extract S, L, and S + L). Table S2. Secondary metabolites identified from Strawberry Fruit (S) extract using LC-MS/MS in ESI-Negative ionization mode. (Metabolites were characterized based on retention time (RT), molecular weight (M.wt), parent ion (*m*/*z*), and diagnostic mass fragments). Table S3. Secondary metabolites identified from Lemon juice (L) extract using LC-MS/MS using ESI-negative ionization mode. (Metabolites were characterized based on retention time (RT), molecular weight (M.wt), parent ion (*m*/*z*), and diagnostic mass fragments). Table S4. Secondary metabolites identified from S/L extract using LC-MS/MS using ESI-negative ionization mode. (Metabolites were characterized based on retention time (RT), molecular weight (M.wt), parent ion (*m*/*z*), and diagnostic mass fragments). Figure S1. Metabolites identified in the strawberry–lemon co-extract (S/L) by LC–ESI–MS/MS operated in negative ionization mode.
