# Supplementary material for: Lemon Juice-Assisted Green Extraction of Strawberry Enhances Neuroprotective Phytochemicals: Insights into Alzheimer’s-Related Pathways
Source: Pharmaceuticals (Basel). 2025 Dec 15;18(12):1892. doi: 10.3390/ph18121892 (PMC12736459; doi:10.3390/ph18121892)
Supplement: Supplementary file 1 [file pharmaceuticals-18-01892-s001.zip › pharmaceuticals-4005514-supplementary.pdf]

Supplementary Material

Lemon Juice–Assisted Green Extraction of Strawberry Enhances Neuroprotective Phytochemicals: Insights into Alzheimer's-Related Pathways

Table S1. HPLC-Based analysis of phenolic compounds identified as area percentage (% Area) (Values represent the relative area percentage of identified phenolic compounds in extract S, L, and S+L)

Compound	S	L	S+L
Gallic acid	686.40	2122.26	5301.79
Chlorogenic acid	3564.36	21660.21	4558.92
Catechin	0	1034.36	52.80
Methyl gallate	25.64	12.61	0
Coffeic acid	55.17	84.87	33.11
Syringic acid	208.05	754.43	129.32
Rutin	275.09	0	156.22
Ellagic acid	98.78	788.65	214.58
Coumaric acid	207.75	36.75	25.80
Vanillin	10.10	15.51	5.81
Ferulic acid	53.04	0	3.95
Naringenin	104.33	13.22	55.18
Rosmarinic acid	79.83	0	12.75
Daidzein	53.04	0	0
Querectin	58.80	4.74	12.42
Cinnamic acid	0	44.69	9.36
Kaempferol	7.11	4.78	0
Hesperetin	21.10	0	0

S: Strawberry extract; L: Lemon-juice extract; S+L: Combined extract (lemon juice–assisted strawberry extract).

Table S2. LC–ESI–MS/MS library of secondary metabolites tentatively identified in strawberry fruit (S) extract in negative ionization mode

Peak No.	Metabolite	Chemical formula	RT (min)	M.wt	[M−H]⁻ (m/z)	Diagnostic MS/MS fragments (m/z)	Library / DB	Library ID / Accession	Match score (%)	Error (ppm)
1	Pentonic acid	$C_5H_{10}O_6$	1.61	166.04	165.03	103.99, 120.96, 136.99, 149.08, 165.03	MassBank	LIB0001	88.5	75.4
2	Gallic acid	$C_7H_6O_5$	1.90	170.02	169.09	124.99, 141.99	MassBank	LIB0002	93.2	60.3
3	2-Deoxyerythropentono-1,4-lactone	$C_5H_8O_4$	2.26	132.04	131.02	102.92, 113.01, 118.96, 129.99, 131.03	MassBank	LIB0003	86.7	82.7
4	p-Coumaric acid	$C_9H_8O_3$	4.72	164.04	163.00	119.04, 146.91	MassBank	LIB0004	94.1	55.1
5	Cyanidin-3-O-hexoside	$C_{21}H_{21}O_{11}$	5.26	449.10	448.94	125.05, 179.05, 259.09, 286.97	MassBank	LIB0005	90.5	68.9
6	Isozonarol	$C_{21}H_{30}O_2$	5.57	314.22	313.04	151.14, 176.95, 194.89, 268.89, 294.62, 312.86	MassBank	LIB0006	84.3	95.2
7	Chlorogenic acid	$C_{16}H_{18}O_9$	6.11	354.09	354.99	134.12, 160.09, 175.07, 193.06	MassBank	LIB0007	92.8	72.5

Peak No.	Metabolite	Chemical formula	RT (min)	M.wt	[M−H]⁻ (m/z)	Diagnostic MS/MS fragments (m/z)	Library / DB	Library ID / Accession	Match score (%)	Error (ppm)
8	Roseoside	$C_{19}H_{30}O_8$	6.99	386.19	385.04	153.10, 205.15	MassBank	LIB0008	87.9	88.6
9	Pelargonidin-3-glucoside	$C_{21}H_{21}O_{10}$	7.04	433.11	415.01	100.91, 161.05, 270.07	MassBank	LIB0009	91.6	79.4
10	Pelargonidin-3-malonylglucoside	$C_{24}H_{23}O_{13}$	9.19	519.42	518.04	136.08, 151.14, 375.07	MassBank	LIB0010	89.7	83.1
11	Hesperidin	$C_{28}H_{34}O_{15}$	9.43	610.19	608.96	301.08, 325.06	MassBank	LIB0011	93.5	64.8
12	Isoquercitrin	$C_{21}H_{20}O_{12}$	9.52	464.09	464.04	109.08, 127.10, 208.19, 223.13, 275.14, 292.19, 301.03, 336.18	MassBank	LIB0012	94.0	59.7
13	α-Bisabolol	$C_{15}H_{26}O$	10.77	222.19	221.10	164.18, 205.21, 220.26, 221.16	MassBank	LIB0013	88.9	90.3
14	Ellagic acid	$C_{14}H_6O_8$	19.71	302.19	301.08	143.19, 217.16, 285.11	MassBank	LIB0014	95.1	57.2
15	Peonidin-3-glucoside	$C_{22}H_{23}O_{11}$	22.28	463.11	461.28	329.08, 461.28	MassBank	LIB0015	90.8	73.9
16	Quercetin 3-xyloside-7-glucoside	$C_{26}H_{28}O_{16}$	26.81	596.14	595.34	435.38, 467.17, 557.26, 595.29	MassBank	LIB0016	92.2	69.5

Table S3. LC–ESI–MS/MS library of secondary metabolites tentatively identified in lemon juice (L) extract in negative ionization mode

ID	Metabolite	Chemical formula	RT (min)	M.wt	Precursor ion (m/z)	Diagnostic MS/MS fragments (m/z)	Library / DB	Library ID / Accession	Match score (%)	Error (ppm)
1	Gallic acid	$C_7H_6O_5$	0.14	170.02	169.01	125.03, 132.96, 150.92	MassBank	LIB0101	93.1	62.3
2	Citric acid	$C_6H_8O_7$	2.41	192.02	174.94	110.95, 118.96, 138.97, 146.99, 159.02, 175.07	MassBank	LIB0102	91.4	70.5
3	Vanillic acid	$C_8H_8O_4$	2.57	168.04	166.99	108.01, 152.03	MassBank	LIB0103	89.8	78.2
4	Coumaric acid	$C_9H_8O_3$	3.35	164.04	163.01	119.04	MassBank	LIB0104	92.7	58.9
5	Citral	$C_{10}H_{16}O$	3.81	152.12	151.04	108.01, 120.97, 135.08	MassBank	LIB0105	88.3	85.1
6	Linalool	$C_{10}H_{18}O$	4.60	154.13	152.98	106.97, 123.01	MassBank	LIB0106	87.5	88.7
7	Eriocitrin	$C_{27}H_{32}O_{15}$	8.16	596.17	594.94	107.03, 151.06, 286.97, 458.99, 594.93	MassBank	LIB0107	90.9	73.4
8	Narirutin	$C_{27}H_{32}O_{14}$	8.90	580.17	578.95	151.04, 271.02, 313.01	MassBank	LIB0108	89.2	79.6
9	Hesperidin	$C_{28}H_{34}O_{15}$	9.33	610.18	609.11	286.05, 301.07, 325.04	MassBank	LIB0109	92.0	65.7

ID	Metabolite	Chemical formula	RT (min)	M.wt	Precursor ion (m/z)	Diagnostic MS/MS fragments (m/z)	Library / DB	Library ID / Accession	Match score (%)	Error (ppm)
10	Apigenin	$C_{15}H_{10}O_5$	10.22	270.05	269.06	123.09, 153.14, 207.15, 251.14	MassBank	LIB0110	90.1	72.9
11	Chlorogenic acid	$C_{16}H_{18}O_9$	10.54	354.09	336.99	120.03, 148.06, 177.02, 234.01, 336.99	MassBank	LIB0111	91.6	69.3
12	Diosmetin	$C_{16}H_{12}O_6$	10.82	300.05	299.05	149.08, 176.08, 255.00, 277.04, 282.96	MassBank	LIB0112	88.9	81.2
13	Kaempferol	$C_{15}H_{10}O_6$	13.20	286.04	285.04	133.10, 193.00, 242.08, 270.00	MassBank	LIB0113	90.5	76.8
14	Limonin	$C_{26}H_{30}O_8$	13.85	470.19	469.03	229.18, 278.12, 321.22, 381.13	MassBank	LIB0114	87.1	89.9
15	Ellagic acid	$C_{14}H_6O_8$	19.63	302.19	301.07	143.18, 217.11, 285.12	MassBank	LIB0115	93.5	60.1

Table S4. LC–ESI–MS/MS library of secondary metabolites tentatively identified in S/L extract in negative ionization mode

ID	Metabolite	Chemical formula	RT (min)	M.wt	Precursor ion (m/z)	Diagnostic MS/MS fragments (m/z)	Library / DB	Library ID / Accession	Match score (%)	Error (ppm)
1	Turanose	$C_{12}H_{22}O_{11}$	1.06	342.11	340.99	100.86, 113.02, 143.07, 161.06	MassBank	LIB0201	89.5	80.4
2	Daphnetin	$C_9H_6O_4$	1.10	178.02	177.00	110.96, 129.02, 148.95, 158.97	MassBank	LIB0202	90.2	76.3
3	4-Methoxy-2,5-dimethyl-3(2H)-furanone	$C_7H_{10}O_3$	1.40	142.06	140.98	110.99, 122.98, 136.85, 139.03, 140.97	MassBank	LIB0203	87.9	83.1
4	D-3-Phenyllactic acid	$C_9H_{10}O_3$	1.74	166.06	165.02	100.92, 118.98, 145.00	MassBank	LIB0204	88.7	79.8
5	O-trans-Cinnamoyl-β-D-glucopyranose	$C_{15}H_{18}O_7$	2.00	310.10	308.96	123.02, 135.09, 141.08, 151.06, 177.11, 245.96	MassBank	LIB0205	91.1	72.4
6	Threitol	$C_4H_{10}O_4$	4.78	122.05	121.05	107.98, 118.90, 121.02, 133.94	MassBank	LIB0206	86.8	88.7
7	Methyl butyrate	$C_5H_{10}O_2$	4.87	102.06	101.06	100.79	MassBank	LIB0207	87.3	90.1
8	2-Methylbutanoic acid	$C_5H_{10}O_2$	5.75	102.06	101.06	100.78	MassBank	LIB0208	88.1	85.9
9	Gallic acid	$C_7H_6O_5$	5.83	170.02	168.89	125.03, 132.96, 150.94	MassBank	LIB0209	92.4	61.2

ID	Metabolite	Chemical formula	RT (min)	M.wt	Precursor ion (m/z)	Diagnostic MS/MS fragments (m/z)	Library / DB	Library ID / Accession	Match score (%)	Error (ppm)
10	Tormentic acid	$C_{30}H_{48}O_5$	7.40	488.34	487.11	147.05, 183.06, 249.02, 374.54, 424.63, 451.24, 487.10	MassBank	LIB0210	89.9	77.3
11	β-Ocimene	$C_{10}H_{16}$	8.32	136.12	135.97	105.95, 117.95, 135.97	MassBank	LIB0211	86.5	89.5
12	Ellagic acid	$C_{14}H_6O_8$	11.88	302.19	301.09	202.08, 229.07, 244.05, 301.09	MassBank	LIB0212	93.0	59.8
13	2-Oxobutyric acid	$C_4H_6O_3$	7.85	102.03	100.95	100.81	MassBank	LIB0213	88.4	82.2
14	7,7-Dimethyl-3,4-octadiene	$C_{10}H_{18}$	7.93	138.14	137.13	137.98, 155.99	MassBank	LIB0214	87.0	86.9
15	Undecane	$C_{11}H_{24}$	8.13	156.18	155.18	136.88, 154.93	MassBank	LIB0215	86.2	92.4
16	Thymidine	$C_{10}H_{14}N_2O_5$	9.06	242.08	241.07	110.98, 154.97, 194.99, 222.89, 240.94	MassBank	LIB0216	90.7	75.1
17	Cyanidin	$C_{15}H_{11}O_6$	13.34	287.05	286.15	118.09, 238.29, 240.33, 242.28, 268.26, 286.18	MassBank	LIB0217	89.3	81.7
18	Chlorogenic acid	$C_{16}H_{18}O_9$	18.06	354.09	353.11	163.19, 177.12	MassBank	LIB0218	91.6	68.9

ID	Metabolite	Chemical formula	RT (min)	M.wt	Precursor ion (m/z)	Diagnostic MS/MS fragments (m/z)	Library / DB	Library ID / Accession	Match score (%)	Error (ppm)
19	Chrysin	$C_{15}H_{10}O_4$	20.94	254.05	253.04	138.06, 152.08, 166.08, 235.06, 253.19	MassBank	LIB0219	90.0	73.5
20	2,4-Bis(1,1-dimethylethyl)-phenol	$C_{14}H_{22}O$	22.98	206.16	205.11	189.26, 205.17	MassBank	LIB0220	87.8	88.1
21	Peonidin-3-glucoside	$C_{22}H_{23}O_{11}$	23.00	463.11	461.25	279.22, 461.24	MassBank	LIB0221	89.7	70.4
22	Pelargonidin-3-malonylglucoside	$C_{24}H_{23}O_{13}$	24.43	519.10	498.07	452.31, 471.27, 498.07	MassBank	LIB0222	88.9	79.2

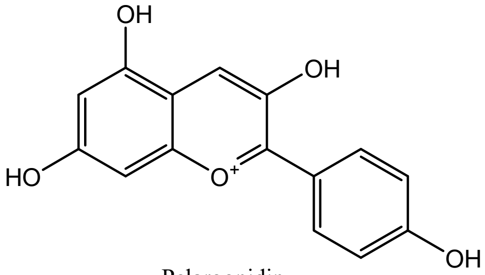

Pelargonidin

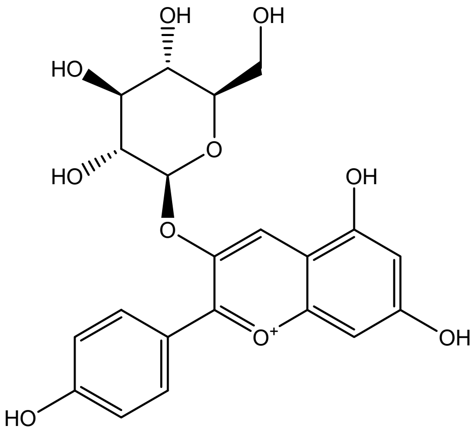

Pelargonidin-3-malonyl-glucoside

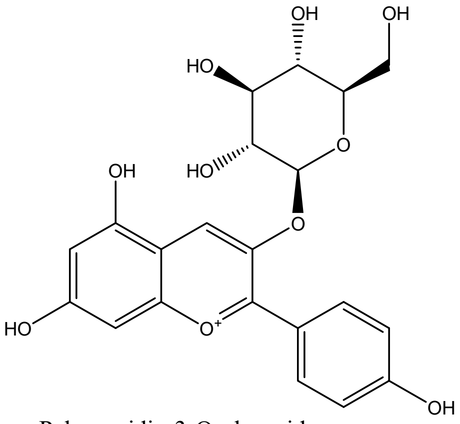

Pelargonidin-3-O-glucoside

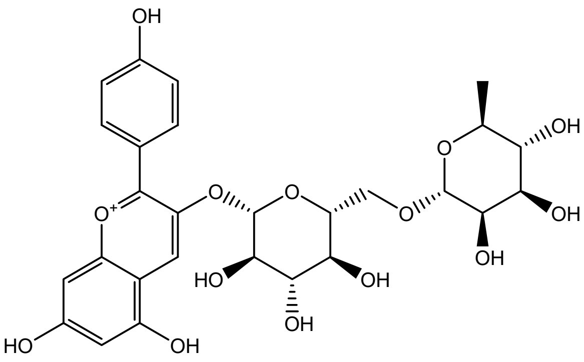

pelargonidin 3-O-rutinoside

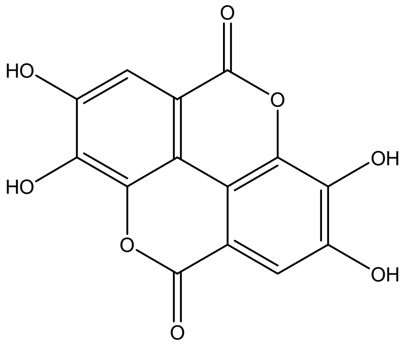

Ellagic acid

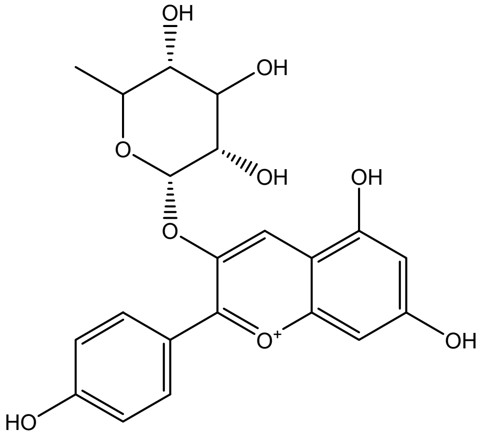

Pelargonidin-3-malonyl-rhamnoside

Figure (S1). Metabolites identified in the strawberry–lemon co-extract (S/L) by LC–ESI–MS/MS operated in negative ionization mode.

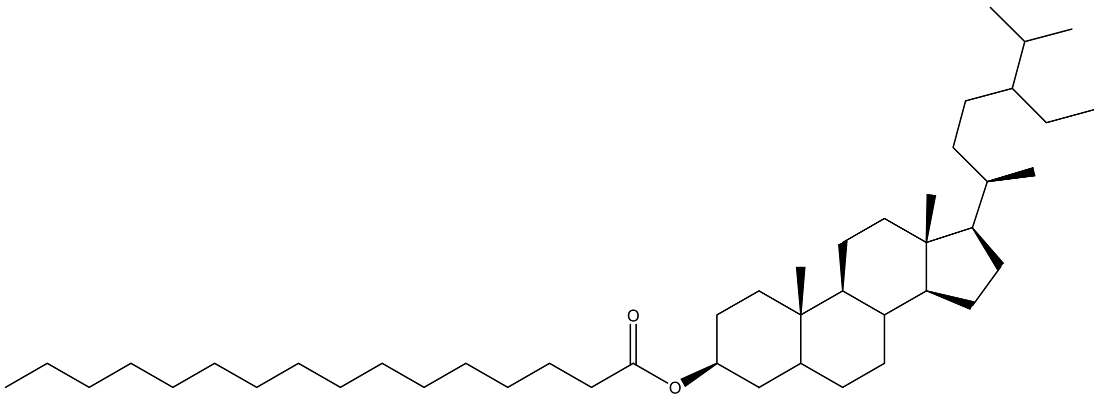

P-Coumaroyl hexose

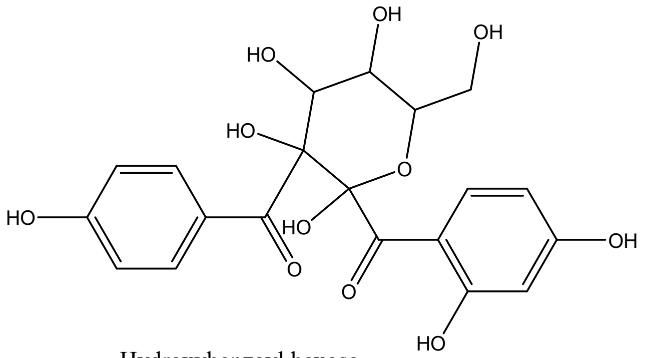

Hydroxybenzoyl hexose

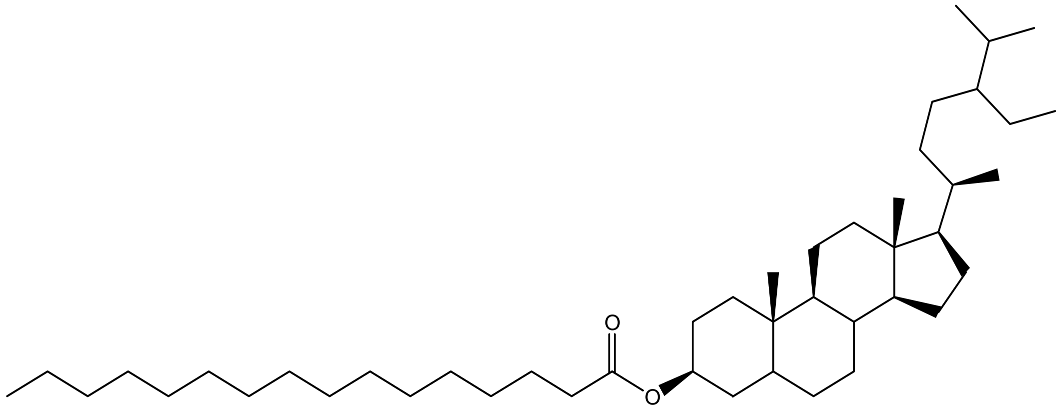

p-Coumaroylhexose-4-O-hexoside

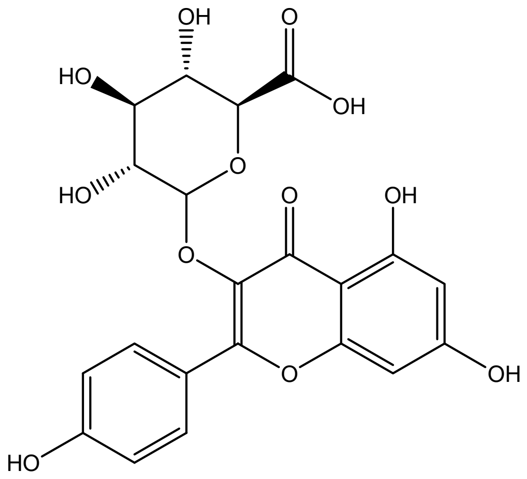

Kaempferol-glucuronide

Continue Figure (S1)

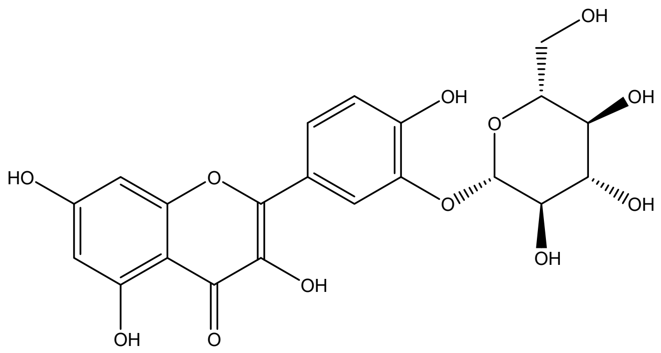

Quercetin-3-O-glucoside

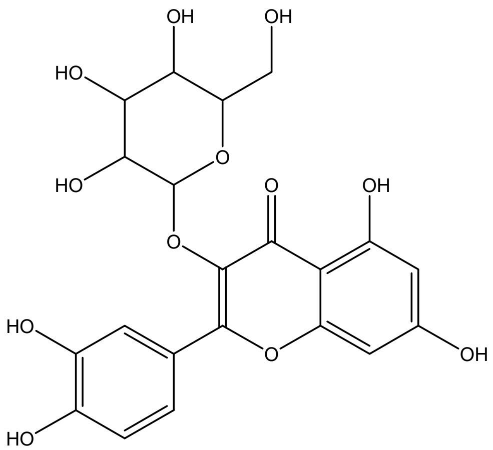

Quercetin-3-O-galactoside

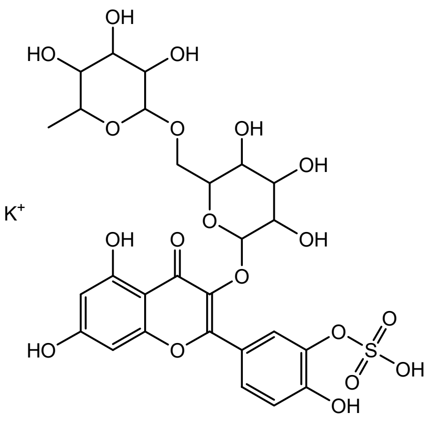

Quercetin-3-O-rutinoside

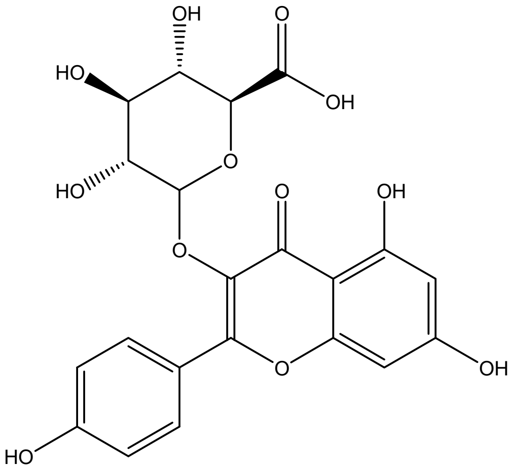

Kaempferol-glucuronide

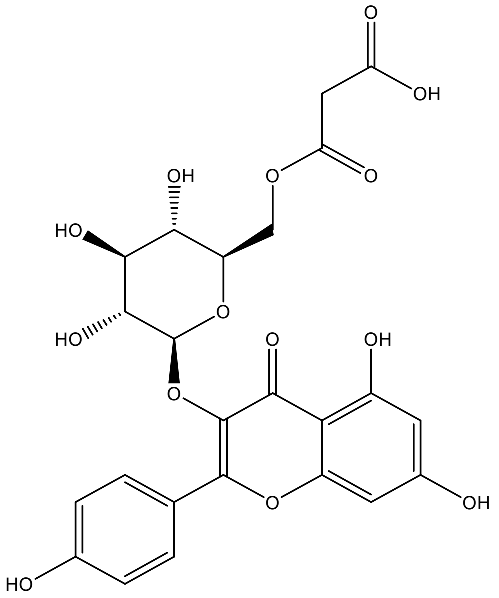

Kaempferol-3-malonylglucoside

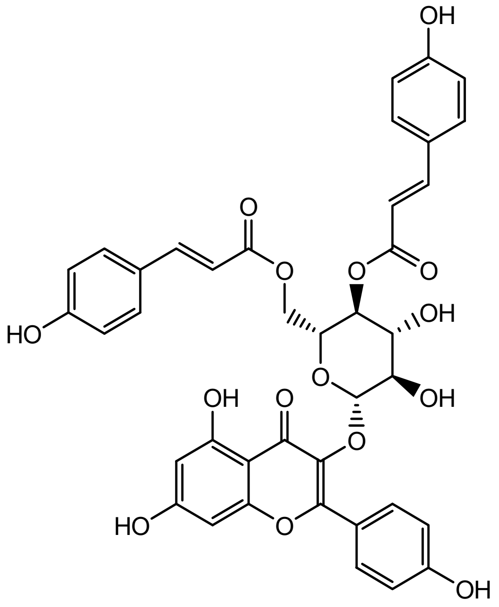

Kaempferol-3-coumaroylglucoside

Continue Figure (S1)

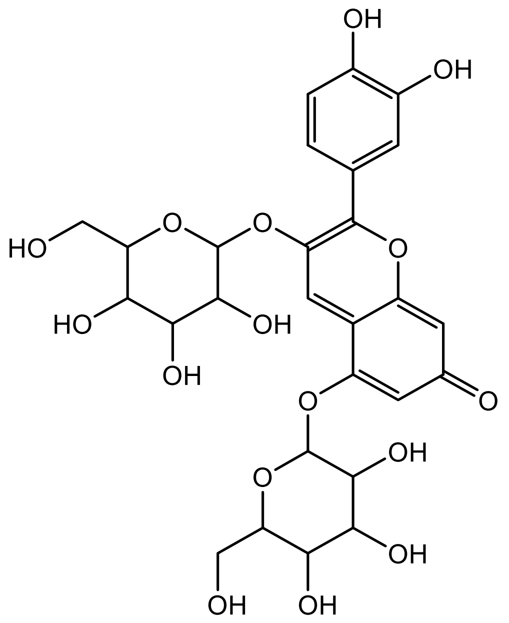

Cyanidin-diglucoside

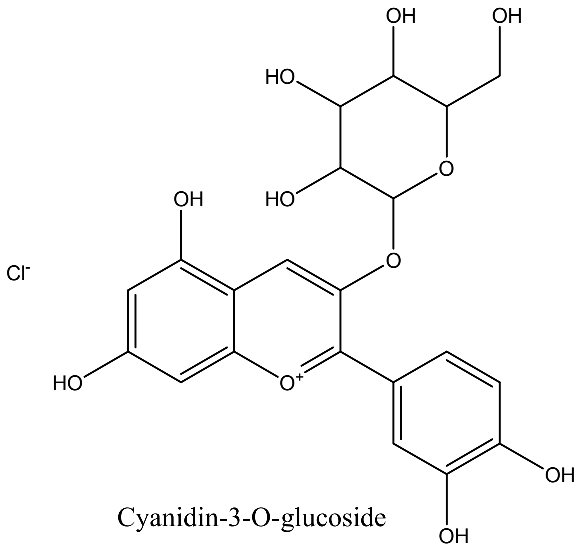

Cyanidin-3-O-glucoside

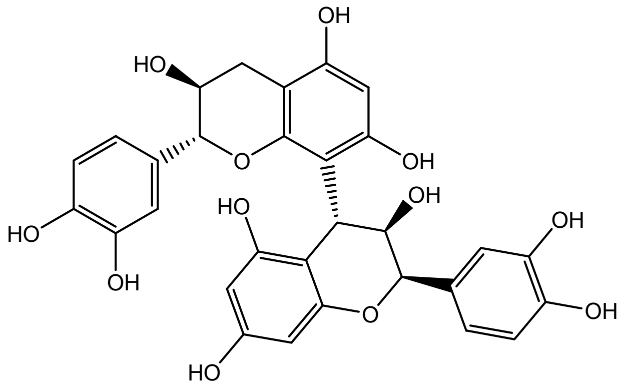

Procyanidin B1

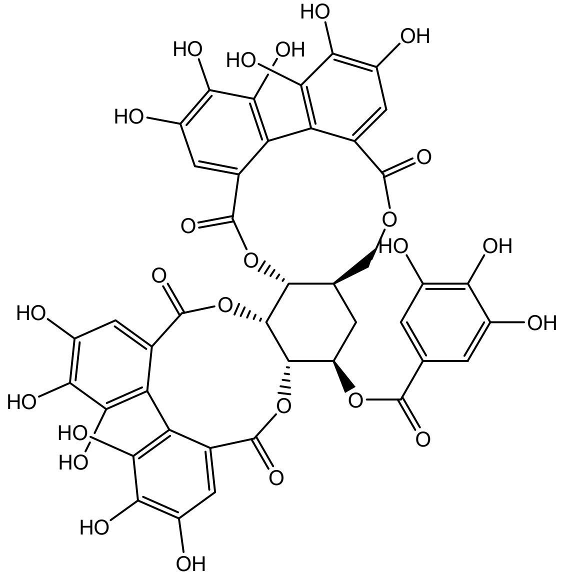

Galloyl-HHDP-glucose

Continue Figure (S1)
